# Drying kinetics and thermodynamic analysis; enhancing quinoa (*Chenopodium quinoa Willd.)* quality profile via pre-treatments assisted germination and processing

**DOI:** 10.1016/j.ultsonch.2025.107337

**Published:** 2025-04-04

**Authors:** Jabir Khan, Palwasha Gul, Qingyun Li, Kunlun Liu

**Affiliations:** a Henan University of Technology, College of Food Science and Engineering, Zhengzhou 450001, PR China; b Henan University of Technology, College of Food and Strategic Reserves, Zhengzhou 450001, PR China

**Keywords:** Quinoa, Pre-treatments, Germination, Drying, Mathematical modeling, Antioxidant activity, Anti-nutritional, Structural properties

## Abstract

Pre-treatments assisted germination is an efficient technique to enhance the nutritional profile of Quinoa (*Chenopodium quinoa Willd.)*. The present study investigated the impact of pre-treatments assisted germination of quinoa nutritional, anti-nutritional, and structural properties. Quinoa grains JQ-778 were subjected to various pre-treatments including soaking, ultrasound at 28 kHz &40 kHz (US 28 kHz, US 40 kHz) for 30 min followed by germination over 96-hour at 25 °C in a Biochemical-Incubator, 12/12 h dark and light dried at temperatures 50 °C, 60 °C, 70 °C, and combined temperatures (70 °C, 60 °C, 50 °C). Among evaluated models, page and logarithmic showed the best fit, presenting the highest, R^2^ ≥ 0.9991, X^2^ ≤ 0.0013, RMSE ≤ 0.0022, and RSS ≤ 0.0201. Moisture diffusion varied from 3.74 × 10^−9^ to 8.36 × 10^−9^, with R^2^ 0.9272 to 0.9837, and energy activation from 18.25 to 28.41 kJ/mol with R^2^ 0.9533–0.9896. US 40 kHz significantly lowered drying time without affecting germinated quinoa grains bioactive components or other qualitative factors. Ultrasonic pre-treatment at 40 kHz and drying at 60 °C yielded the highest antioxidant potency composite index of 98.78 %. The content of phytic acid and tannin dropped by 66.66 to 82.99 % and 31.48 to 41.60 %, respectively (p < 0.05). Each treatment significantly altered quinoa’s quality attributes. Principal Component Analysis revealed significant correlations between analyses, explaining 80.37 % variability. The intensity of functional groups decreased in the infrared spectra, although the transmission of signals was greater in pretreated samples than in control. Scanning electron microscopy analysis showed extensive fragmentation and surface erosion of quinoa grains after ultrasound treatment. Our data suggests that ultrasound-treated quinoa grains may enhance their nutritional value, making them a suggested source of high-protein grains, bioactive components, with distinct structural properties.

## 1 Introduction

Quinoa, an Andean grain (*Chenopodium quinoa Willd.*), has gained popularity globally owing to its high nutritional value and balanced nutrient content [1]. Protein content ranges from 16.5-19 % of high biological value [2,3], crude fibre 1.92 to 3.38 %, ash (2.21 to 2.43 %) [4], carbohydrates 68.8 to 75.82 % [5,6], and energy (331–381 kcal/g) [7]. A biotechnological process, controlled germination enhance the nutritional profile of Andean grains [8,9]. Several metabolic pathways are triggered during germination, allowing stored nutrients to be mobilized, producing new bioactive molecules as well as lowering anti-nutritional agents [10,11].

In this context, food processing techniques like soaking and ultrasound pre-treatments followed by germination had a significant impact on grains. Several investigations have found alterations in protein quality after the use of such processing techniques [12,13]. Germination process may regarded as a good strategy for enhancing the nutritional value of grain grains [14]. Ultrasound pre-treatments assisted germinated quinoa grains has been found significantly increased total flavonoid and phenolic content, potentially enhancing its nutritional properties [15,16]. Previous studies suggested, that soaking is a traditional method for quinoa grains germination. In modern era pre-treatments have been used to increase the quality of germinated grains [17,18]. However, each pre-treatment may affect grain quality differently [19]. The application of ultrasound pre-treatments to enhance the quality of grains has been recently explored in scientific studies, including quinoa [20], rice [21], and wheat [16]. However, the impact of ultrasound pre-treatments at different frequencies followed by controlled germination, drying at different temperatures on nutritional and anti-nutritional factors has not been investigated yet. Grains profile must be studied in order to verify whether the treatment has affected properties of grains.

A variety of drying techniques are employed to preserve food materials, including hot air drying, Sun energy, microwave, infrared and other. Hot air drying, a traditional and widely employed method, is a relatively energy-intensive strategy that has been utilized for centuries [[22], [23], [24]]. It also includes mass and heat transfer at the same time as well as a phase shift. The drying process is gaining popularity again due to the need for high-quality fast-dried goods. Another emerging trend is the integration of non-destructive methods with drying technologies to optimize efficiency and minimize quality degradation [25]. This technique involves soaking samples in hypertonic water or water before germination and drying. It removes moisture without raising the temperature, therefore food is less harmed than with typical drying [26]. Ultrasonic waves may increase heat-mass transfer by changing boundary layers, material diffusivity and density [27].

Several scientific studies have highlighted the health benefits of quinoa grains [[28], [29], [30]]. According to El-Seedi et al. [31], bioactive components such dietary fiber, flavonoids, and polyphenols are responsible for these advantages. Although quinoa has several potential uses, it may be limited due to anti-nutritional components including phytic acid and tannins [7]. According to recent published studies, Mohanto et al. [32] and Patiballa et al. [33], germination decreases the quantity of anti-nutritional elements such phytic acid while increasing the amount of antioxidant properties. Despite some knowledge about the anti-nutritional agents of quinoa, most studies are concentrated on saponins [34]. In contrast, no research has examined the effects of different pretreatments of quinoa grains on the phytic acid and tannins content. No published studies have examined the effects of sequential ultrasonic treatment on quinoa grains quality profile that have been germinated in a controlled Biochemical Incubator for 96 h using a 12/12 dark and light cycle followed by drying at different temperatures. Additionally, the effectiveness of combination techniques has not been evaluated in any studies. Hence, this study aim to address a gap in the current literature by studying the effects of various pretreatments on quinoa grains, including soaking, ultrasonic sound at 28 kHz and 40 kHz for 30 min assisted germination, with an emphasis on mathematical modeling, thermodynamics, nutritional composition, colour values, bioactive compounds, enzymatic activities, anti-nutritional and structural properties.

## 2 Material and methods

### 2.1 Germination

**Fig. 1:**
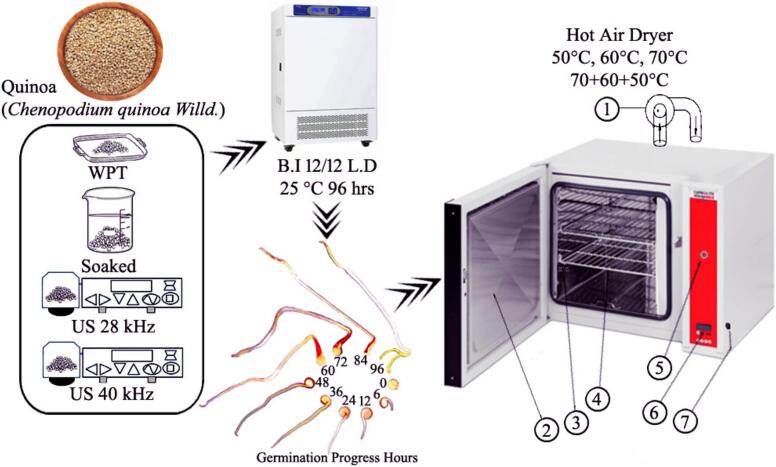
Plan of the study, Biochemical Incubator (B.I); Light and dark circle (L.D); Ultrasound 28 kHz (US 28 kHz); Ultrasound 40 kHz (US 40 kHz); Centrifugal pan (1); Door (2); Temperature detector (3); Chamber trays (4); Air controller (5); Screen (6); Switch (7).

Quinoa grains JQ-778 were purchased from (Jingle Branch Shanxi China); 400 ± 2 g of grains were measured for each experimental conditions and washed four times in a 2:1, while shaking at 100 rpm for 5 min. After draining the water, the grains were separated into four groups: without pre-treated (WPT), Soaked, US 28 kHz, and US 40 kHz. For WPT, quinoa grains were kept for germination directly after washing, while for soaking, grains were dipped in distilled water (DW) with 1:4 (w/v) for 2 h. An ultrasonic bath generator were used for Ultrasound pre-germination treatments 28 kHz, 40 kHz with 100 % amplitude, in which quinoa grains were steeped in DW 1:4 (w/v) for 30 min at 25 ± 2°C. Trays were used to germinate all the quinoa samples at 25 °C in Biochemical Incubator (Model: SPX-250B-Z11) for 12/12 h dark and light circle. Pictures of germinated states were taken at 0, 6, 12, 24, 36, 48, 60, 72, 84, and 96 h (Fig. 1). Germinated sample was dried in Hot-Air Dryer (Model: S202305103) at different temperatures, 50 °C, 60 °C, 70 °C, and combined temperatures (4 h on 70 °C, 4 h on 60 °C, and finally on 4 h on 50 °C up to constant weight). Combined temperatures was based on mean time hours from each temperature. Each experiment was conducted in replicate, the resulting dried sample were milled and stored in refrigerator for further analysis. Several drying models (Newton, Logarithmic, Two-term, Page, Handerson & Pabis and Modified Henderson & Pabis) were employed on dried samples to analyse the drying kinetics and understand their drying behaviours.

### 2.2 Drying

The germinated quinoa grains were dried in Hot-Air Dryer at different temperatures and the velocity of dryer were controlled at1.5 m/s, measured by an anemometer. An experimental design included 17 trials included all germinated groups and control. First Hot air dryer was run for 30 min to reach stable stat. After that, for each conducted experiment in the drying chamber, the trays loaded density was 100 ± 2 g. In order to determine their weight loss on an analytical scale, samples were weighed hourly until achieved the target moisture content until the weight of each trays sample remain constant. Immediately after each weighting, the samples were kept back in the dryer.

### 2.3 Drying kinetics

#### 2.3.1 Mathematical modelling

A mathematical modelling approach were employed to study the drying behaviours of germinated quinoa grains. Moisture ratio was calculated using an empirical model for all samples. Moisture ratio was calculated using (**Eq.** (1) and for drying rate (**Eq.** (2) were used.

(1) $$\mathrm{MR}=\frac{\mathrm{Mt}-Me}{\mathrm{Mo}-Me}$$

*MR* denote moisture ratio, *Mt*, Mo, Me = initial, equilibrium, and initial moisture content.

(2) $$\mathrm{DR}=\frac{Mt1-Mt2}{t1-t2}$$

*Mt*1 and *Mt*2 represent the initial and final moisture contents, while *t*1 and *t*2 denote the corresponding drying times.

#### 2.3.2 Determination of effective moisture diffusivity

Moisture diffusivity of dried quinoa grains were calculated using (**Eq.** (3).

(3) $$\mathrm{MR}=\frac{\mathrm{Mt}-Me}{\mathrm{Mo}-Me}=\frac{8}{\pi^{2}}exp\left(\frac{-\pi^{2}Defft}{{4L}^{2}}\right)$$

*Deff* obtained from the slope (K) which is associated with (**Eq.** (4) of the linear regression between ln(MR) and drying time (t).

(4) $$K=\frac{\pi^{2}Deff}{{4L}^{2}}$$

*Deff* was expressed in m^2^/s, while L represent half the mean thickness 0.0015 for the quinoa grain mean of slab (m).

### 2.4 Extract preparation

The extraction from the quinoa powder was done by following the protocols of Enciso-Roca et al. [35], with slight modification. Control and pre-treated sample powder (0.5 g) was homogenised in 5 mL 80 % ethanol. The mixture was subjected to vigorous agitation in a shaking incubator (Multitron II Infors SARL, Massy, France) for 30 min at 160 rpm, the homogenized was then centrifuged (Centurion K1240) at 10677 × g for 10 min. The supernatant was carefully removed. The process were repeated again from the residue under the same conditions. The extract were combined and passed through nylon filter 0.45 μm and kept at 4 °C for further analysis.

### 2.5 Proximate

Proximate of all conditions quinoa samples were determined according to the standard method of the Association of Official Analytical Chemists (AOAC) 2005 [36] for moisture; the moisture content analyser (Denver instrument IR-30) were used. For protein; kjeldahl (Hanon, K1160) with 6.25 protein factor, for fat petroleum ether in a Soxhlet extractor were used. Ash content was determined in muffle furnace & fiber was determined by ignition of the dry residue after digestion with sulphuric acid and sodium hydroxide. Carbohydrate content was determined by using the following formula; Carbohydrate % = 100 % – (%moisture + %fat + %protein + %fibre + %ash). For energy, protein and total carbohydrate were multiplied by 4 kcal/g, whereas fat was multiplied by 9 kcal/g [37]. Each analysis was performed in replicates and reported as percentages.

### 2.6 Colour

A digital colorimeter (CR-5, Konica Japan) was used to measure colour coordinates L*, a* & b* (brightness from black to white; green to red; blue to yellow), using a standard illuminant D65 and a 10° observer and data were taken in reflectance modulus. Change in colour differences (ΔE) were calculated using ΔE = ((ΔL^*2^ + Δa^*2^ + Δb^*2^) ½) [38]. Hue angle (H) were calculated by using H = tan^−1^ (b*/a*) when a* > 0 and b* > 0; Chroma C*= (a^*2^ + b^*2^) ^½^ [39]; Browning Index; BI = 100(*X*_a_-0.31)/0.17, whereas (*X_a_* = a*+1.75l*/5.645 l*+a*0.301b*) [40].

### 2.7 Total flavonoid & phenolic content (TFC &TPC)

TFC were evaluated using Jogihalli et al. [41] technique with only minor modifications. In short, 0.25 mL quinoa extract was mixed with 0.15 mL distilled water and 0.15 mL NaNO_2_ (5 g/100 mL) followed by incubation for 6 min. Following which 0.15 mL AlCl_3_ (10 g/100 mL) aqueous solution was added. After 6 min 0.15 mL of 1 mol/L NaOH was added to the reaction mixture followed by immediate addition of 2 mL distilled water. The content were mixed thoroughly and was measured at 510 nm by Spectrophotometer (GC/FT-IR, WI. 5371, Made in USA). Quercetin's calibration curve was created (y = 0.0018x −0.0469, R^2^ = 0.997) and data were reported as mg QE/100 g.

TPC content was measured using Folin-Ciocalteu reagent by following the protocols of Lagnika et al. [42]. Briefly, 0.5 mL of quinoa extract were mixed with 1 mL of acidified methanol and 5 mL of Folin-Ciocalteu reagent (1:10 dilution with distilled water). After that, 4 mL of NaHCO_3_ solution was added and the content were vortexed followed by incubation for 2 h for complete reaction. Using a Spectrophotometer (GC/FT-IR, WI. 5371, Made in USA), the absorbance was measured at 765 nm against a reagent blank. Gallic acid calibration curve was created (y = 0.0019x + 0.0446, R^2^ = 0.977), data were expressed in mg GAE/100 g.

### 2.8 Antioxidants activities

#### 2.8.1 DPPH radical scavenging activity

DPPH scavenging was assessed using Singh et al. [43] technique with slight modifications. First, 0.1 mL quinoa extract was combined with 4.9 ml DPPH (freshly prepared solution, 0.1 mmol/L in methanol). The mixture was incubated for 30 min at 25 °C in a dark at room temperature. Absorbance at 517 nm were measured by 0 and 30 min using methanol blank. The DPPH scavenging activity was calculated using the following formula DPPH (%) = (A_control_ – A_sample_)/A_control_ × 100.

#### 2.8.2 FRAP

FRAP was assessed using Benzie and Strain, [44] with slight modifications. FRAP reagent was made by combining 300 mmol/L acetate buffer (pH 3.6), 10 mM TPTZ solution in 40 mmol/L HCl, and 20 mmol/L FeCl_3_ in a ratio of 10:1:1 (v/v). Briefly, 0.1 mL sample extract and 2.4 mL FRAP reagent were incubated at 37℃ for 10 min at room temperature. The absorbance was measured at 593 nm immediately against reagent blank. Trolex calibration curve was created (y = 0.4545x + 0.0259, R^2^ = 0.9984.), data were reported as μmol TE/g.

#### 2.8.3 ABTS

ABTS radical scavenging activity was examined with minor modifications by using the protocols of Sant'Anna et al. [45]. In short, 20 mM ABTS & 2.45 mM potassium persulfate (1:1v/v) were combined and incubated for 14–16 h in the dark at room temperature before use. The prepared solution were checked at 753 nm absorbance by spectrophotometer to achieve the ABTS^•^ working solution with an absorbance (0.700 ± 0.020) by diluting the stock solution with ethanol (1:22). Then, 1 mL of ABTS^•^ working solution, 0.1 mL of quinoa extract, 3.9 ml ethanol was added and the absorbance was recorded at 734 nm after 6 min against blank.. Results were reported as μmol TE/g.

#### 2.8.4 Reducing power (RP)

RP was determined using the method of Oyaizu [46]. In short, 1 mL of quinoa extract, 2.5 mL of potassium buffer (0.2 mol/L, pH 6.6) and 2.5 mL of 1 g/100 mL potassium ferricyanide (K_3_Fe(CN)_6_) solution were mixed thoroughly and the reaction mixture was incubated at 50 °C for 30 min. After that 2.5 mL of 10 g/100 mL trichloroacetic acid solution was added and the mixture were centrifuged at 1000 × g for 10 min. After centrifugation, 2.5 mL supernatant was mixed with 2.5 mL distilled water and 0.5 mL FeCl_3_ (100 mg/100 mL). Absorbance was read at 700 nm against water as a blank and the results were expressed as mg AAE/g.

#### 2.8.5 Metal chelating activity (MCA)

MCA was measured with a slight modification as reported by Sant'Anna et al. [45].In short, 20 μL of quinoa extract, 50 μL FeSO_4_ and 5 mL distilled water was added. The reaction was initiated by the addition of 75 μL of ferrozine (5 mmol/L). The content were mixed and incubated for15 min at room temperature to reach equilibrium, absorbance was measured at 562 nm against an acidified methanol blank & the results were calculated using MCA (%) = (1- A_sample_ /A_control_) × 100.

#### 2.8.6 Antioxidant potency composite index (APCI)

Overall APCI was calculated by averaging the standardized scores of five antioxidant assays (DPPH^•^, FRAP, ABTS^•^, RP & MCA) [5]. Each assay was given equal weight, with the highest score set at 100. The APCI was calculated as the mean of the standardized scores across all five assays.

### 2.9 Polyphenol oxidase (PPO) and peroxidase (POD)

Extraction procedure for PPO and POD activities were determined using the methods of Teoh et al. [47] technique. Quinoa grain powder 5 g was homogenized in phosphate buffer (30 mL, 0.1 M & adjusted pH 6.2) together with 1 g of polyvinylpyrrolidone, and centrifuged at 3500 rpm for 15 min. The supernatant was collected for determination of PPO and POD activities.

Similar to Sarpong et al. [48], the PPO was assessed. In short, the collected supernatant 0.5 mL was combined with 2 mL of phosphate buffer with adjusted pH 6.2 and 0.1 mL of 100 mM 4-methycatechol. The absorbance were continuously measured at 420 nm for 5 min and expressed as units of enzymatic activities. One unit of PPO activity was defined as the amount of the enzyme that caused a change in absorbance per minute.

The methodology for measuring POD activities was based on that of Zhang et al. [49]. POD activity was measured by mixing previously prepared extract 0.1 mL with 0.1 mL 4 % guaiacol (v/v), 0.1 mL of 1 % H_2_O_2_ (v/v), 2.66 mL phosphate buffer. POD activity were determined by measuring absorbance at 470 nm for 5 min. One unit of PPO activity was defined as the amount of the enzyme that caused a change in absorbance per minute.

### 2.10 Anti nutritional factors

#### 2.10.1 Phytic acid (PA)

A colorimetric method was used to assess PA concentration, following the protocols of Reason et al. [50]. In short, the PA was extracted with 0.2 g quinoa powder, (0.2 N) HCL solution allowed to stand for 2 h at room temperature. The extracts were treated with FE III solution following by centrifugation at 7000 × g for 10 min (Cene, H1750R). The absorbance of the supernatant was measured at 519 nm by spectrophotometer (GC/FT-IR, WI. 5371, USA) against distilled water as a blank. PA concentration was calculated by calibration curve of standard phytic acid solution and results was expressed as mg/g of sample.

#### 2.10.2 Tannin

Tannin content was determined using the vanillin assay as described Thakur et al. [18]. The working solution was prepared by mixing equal amount of methanol solution, 1 % (w/v) vanillin, 70 % sulfuric acid (w/w) just before the reaction. 0.1 mL of quinoa extracts and 0.2 mL of working solution were mixed and incubate at 30 °C for 20 min and absorbance was measured at 500 nm. Catechin as external standard was used for quantification of tannin content and results were expressed as mg catechin equivalent (mgCE/g).

### 2.11 Structural properties

#### 2.11.1 FTIR

Functional group analysis was performed using FTIR. Spectra were obtained using a Nicolet IS50 FTIR transmission module (ThermoScientific, USA) with the following parameters: 1:100 quinoa gtains powder with KBR (potassium bromide), 32 scans, and 8 cm^−1^ resolution [1,51]. All spectra were taken at room temperature.

#### 2.11.2 SEM

All samples microstructure including (Control, WPT, Soaked US 28 kHz, and US 40 kHz) pre-treated before germinated dried at various temperatures was detected using a SEM (FEI, Quanta 250FEG) at the Henan University of Technology by following protocols of Ahmed et al., [52]. Quinoa grains powder was placed on a SEM stub using double-sided tape putter-coated with gold (Ted Pella 108auto) & studied at 3.00 kV with 4000x magnification (scale bar: 40 µm). Cross-section 120x, 1 mm; starch granules and protein bodies, 4000x, 40 µm scale bar; embryonic root, 4000x, 40 µm scale bar; germinated quinoa grain shape, 75x, 2 mm scale bar.

### 2.12 Statistical analysis

Analysis were done in replicate & data were summarized in means ± standard deviations. One Way analysis of variance and a Tukey's test was use for analysis (p < 0.05). Significant differences were denoted by distinct letters. PCA and correlation heat maps were applied to find the effects of pre-treatments assisted germination followed by drying at different temperatures on targeted parameters and other response variables. All analyses & drawing were per formed by IBM SPSS Statistics Version 23.0 software and Origin 2021, respectively.

## 3 Results and discussions

### 3.1 Mathematical modelling

#### 3.1.1 Moisture ratio vs. Drying time

**Fig. 2:**
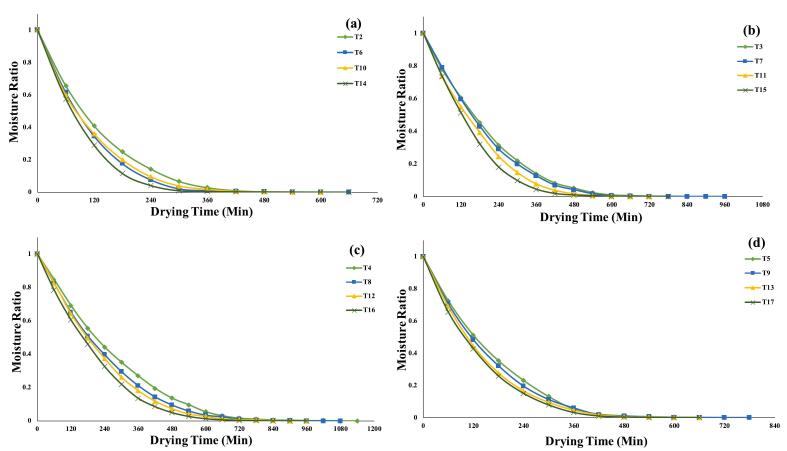
**(a-d).** Curves represent the weight loss of germinated quinoa dried at. 50 °C, (a); 60 °C, (b); 70 °C, (c); and combined temperatures (d).

Experimental data from dried quinoa grains at various temperatures were fitted into thin-layer drying models using nonlinear regression statistics. To precisely represent a drying process, the coefficient of determination (R^2^) must be greater than 0.99 & X^2^ RMSE & RSS, should be low as possible [53]. Seven models exhibited R^2^ values more than 0.99, whereas Thompson, moisture diffusion, and other models were excluded from details discussions, that were either not fitted by our data or had somewhat lower R^2^ values. Depending on the pre-treatment group, the average time to achieve a consistent weight of dried germinated quinoas grains at 50, 60, 70 °C and combination temperatures varied. WPT ranged from 600 to 960 min, soaking 540 to 900 min, US 28 kHz 420 to 840 min and US 40 kHz 480 to 840 for **(**Fig. 2 **a-d**). The time required for achieving a constant weight at 50 °C was double that of sample dried at 70 °C.

According to Luisetti et al. [54], there was a comparable pattern of drying, who analysed germinated quinoa grains (*Chenopodium quinoa Willd.*), and the features of hot air drying temperatures between 40 °C and 80 °C. This study found an inverse association between temperature and drying time, which is comparable with our study. Moscon et al. [55], studied quinoa (*Chenopodium quinoa Willd.*) grain drying kinetics from 40 °C to 70 °C. The author found High temperatures accelerated drying, shortening time and increasing water removal. Other authors have shown comparable results when drying of seeds, fruits, and grains [[56], [57]]. Quinoa grains ultrasonically pre-treated at 28 and 40 kHz and dried at all temperatures showed a reduction drying time and moisture content. Similar to Rashid et al. [57], drying temperatures and ultrasonic intensity lowered drying time and moisture content. Ultrasonic waves cause cavitation, which causes substantial material texture changes, as a result transform product’s structure into sponge-like tissue, which accelerates moisture removal [58].

#### 3.1.2 Validation of mathematical modeling

Several mathematical models were assessed in order to get a better knowledge of the drying kinetics of the germinated quinoa grains dried at different temperatures. Nonlinear regression was used in all models to determine the value of each parameter. The calculated parameters and statistical analysis of the models evaluated for pre-treatments and drying temperatures are shown in (Table 1). To evaluate the quality of the fitted models, we used statistical metrics like R^2^, X^2^, RMSE, and RSS. A model was considered to have satisfactorily represented the drying process if it had a R^2^ value greater than 0.99 & X^2^ RMSE and RSS as low as possible.

**Table 1:** Statistics of applied drying thin-layered models on pre-treated assisted germinated quinoa grains dried under hot air dryer at various temperatures.

**Models**	**50 °C**						**60 °C**					
	**N/O**	**Const. & Coffi.**	**R^2^**	**X^2^**	**RMSE**	**RSS**	**N/O**	**Const. & Coffi.**	**R^2^**	**X^2^**	**RMSE**	**RSS**
	T2	k = 0.31438	0.9981	0.0015	0.0022	0.0019	T3	k = 0.84200	0.9987	0.0002	0.0012	0.0003
**Newton**	T6	k = 0.33408	0.9983	0.0012	0.0019	0.0012	T7	k = 0.38929	0.9987	0.0001	0.0011	0.0003
	T10	k = 0.32093	0.9992	0.0017	0.0014	0.0015	T11	k = 0.41253	0.9991	0.0014	0.0022	0.0024
	T14	k = 0.38043	0.9983	0.0003	0.0019	0.0011	T15	k = 0.50908	0.9982	0.0003	0.0014	0.0012
	T2	a = 0.37812 k = 0.33373 c = 0.01045	0.9994	0.0008	0.0016	0.0014	T3	a = 0.36959 k = 0.41665 c = 0.03706	0.9986	0.0005	0.0016	0.001
**Logarithmic**	T6	a = 0.38359 a = 0.35926 c = 0.00985	0.9988	0.0008	0.0026	0.0022	T7	a = 0.40546 k = 0.41223 c = 0.09323	0.9983	0.0008	0.0016	0.0012
	T10	a = 0.41558 k = 0.33523 c = 0.10718	0.9993	0.0022	0.0021	0.0018	T11	a = 0.41681 k = 0.41527 c = 0.10355	0.9995	0.0022	0.0024	0.0019
	T14	a = 0.41065 k = 0.39084 c = 0.09982	0.9996	0.0012	0.0028	0.0021	T15	a = 0.41065 k = 0.39083 c = 0.09982	0.9996	0.0012	0.0011	0.0009
	T2	a = 0.39791 k 1 = 0.3018 k 2 = 0.29758	0.9984	0.0026	0.0016	0.0018	T3	a = 0.44335 k 1 = 0.37830 k 2 = 0.38971	0.9985	0.0023	0.0021	0.0023
**Two Term Exponential Model**	T6	a = 0.41258 k 1 = 0.30180 k 2 = 0.31987	0.9981	0.0027	0.0032	0.0034	T7	a = 0.43779 k 1 = 0.39622 k 2 = 0.41498	0.9983	0.0022	0.0019	0.0021
	T10	a = 0.44032 k 1 = 0.32220 k 2 = 0.31557	0.9985	0.0029	0.0015	0.0016	T11	a = 0.46635 k 1 = 0.38821 k 2 = 0.42695	0.9985	0.0027	0.0034	0.0035
	T14	a = 0.44253 k 1 = 0.37328 k 2 = 0.38607	0.9977	0.0014	0.0036	0.0035	T15	a = 0.45933 k 1 = 0.42520 k 2 = 0.47768	0.9976	0.0021	0.0024	0.0024
	T2	k = 0.62629 n = 0.56520	0.9994	0.0012	0.0016	0.0012	T3	k = 0.72449 n = 0.53664	0.9993	0.002	0.0011	0.0009
**Page Model**	T6	k = 0.68194 n = 0.54000	0.9998	0.0019	0.0014	0.0019	T7	k = 0.75069 n = 0.57763	0.9991	0.0012	0.0017	0.0019
	T10	k = 0.67897 n = 0.52335	0.9997	0.0025	0.0017	0.0019	T11	k = 0.78608 n = 0.55269	0.9992	0.0014	0.0014	0.0009
	T14	k = 0.72791 n = 0.54958	0.9993	0.0019	0.0019	0.0009	T15	k = 0.82678 n = 0.56798	0.9993	0.0013	0.0009	0.0026
	T2	a = 0.76341 k = 0.33449	0.9994	0.0003	0.0016	0.0017	T3	a = 0.81895 k = 0.44578	0.998	0.0263	0.0036	0.0031
**Henderson & Pabis Model**	T6	a = 0.81942 k = 0.39527	0.9988	0.0115	0.0024	0.0029	T7	a = 0.83905 k = 0.50395	0.9993	0.0016	0.0019	0.0017
	T10	a = 0.81934 k = 0.37598	0.9982	0.0152	0.0025	0.0026	T11	a = 0.85346 k = 0.49676	0.9987	0.0098	0.0036	0.0032
	T14	a = 0.83651 k = 0.46361	0.9988	0.0307	0.0024	0.0025	T15	a = 0.84996 k = 0.56351	0.9988	0.0209	0.0039	0.0034
	T2	a = 0.80829 b = 0.17447 c = 0.15816 k = 0.55900 g = 0.47618 h = 0.03736	0.9994	0.0021	0.0026	0.0024	T3	a = 0.75295 b = 0.16997 c = 0.14736 k = 0.68131 g = 0.46106 h = 048550	0.9989	0.0007	0.0025	0.0025
**Modified Henderson & Pabis Model**	T6	a = 0.79231 b = 0.17296 c = 0.14985 k = 0.60503 g = 0.47518 h = 0.03989	0.9984	0.0023	0.0027	0.0021	T7	a = 0.71678 b = 0.16830 c = 0.13072 k = 0.69273 g = 0.47204 h = 0.04620	0.9986	0.0012	0.0014	0.0015
	T10	a = 0.77594 b = 0.17161 c = 0.15791 k = 0.64908 g = 0.47377 h = 0.04476	0.9983	0.0024	0.0029	0.0028	T11	a = 0.71710 b = 0.16680 c = 0.14010 k = 0.69708 g = 0.43754 h = 0.05753	0.9982	0.0041	0.0024	0.0031
	T14	a = 0.76774 b = 0.17038 c = 0.14160 k = 0.66577 g = 0.47026 h = 0.04573	0.9991	0.0038	0.0024	0.0027	T15	a = 0.71462 b = 0.16373 c = 0.12054 k = 0.69504 g = 0.44041 h = 0.05864	0.9991	0.0049	0.0028	0.0028
**Model Names**	**70 °C**						**Combined temperatures**					
	**N/O**	**Const. & Coffi.**	**R^2^**	**X^2^**	**RMSE**	**RSS**	**N/O**	**Const. & Coffi.**	**R^2^**	**X^2^**	**RMSE**	**RSS**
	T4	k = 0.54876	0.9996	0.0029	0.0024	0.0028	T5	k = 0.44955	0.9992	0.0013	0.0024	0.0021
	T8	k = 0.62110	0.9988	0.0019	0.0009	0.0021	T9	k = 0.50060	0.9982	0.0022	0.0024	0.0019
**Newton**	T12	k = 0.64058	0.9984	0.0021	0.0023	0.0021	T13	k = 0.55718	0.9987	0.0022	0.0024	0.0026
	T16	k = 0.74080	0.9978	0.0032	0.0028	0.0026	T17	k = 0.57976	0.9982	0.0031	0.0029	0.0027
	T4	A = 0.30133 k = 0.42602 c = 0.07410	0.9983	0.0004	0.0029	0.008	T5	A = 0.37731 k = 0.57612 c = 0.09862	0.9995	0.0003	0.0037	0.002
	T8	A = 0.40655 k = 0.60265 c = 0.08750	0.9994	0.0009	0.0018	0.0021	T9	A = 0.40898 k = 0.44390 c = 0.09046	0.9991	0.0019	0.0018	0.0017
**Logarithmic model**	T12	A = 0.44878 k = 0.57374 c = 0.09271	0.9998	0.0018	0.0028	0.0025	T13	A = 0.44848 k = 0.57374 c = 0.09271	0.9996	0.0015	0.0016	0.0012
	T16	A = 0.40810 k = 0.45658 c = 0.08936	0.9991	0.0012	0.0026	0.0011	T17	A = 0.46349 k = 0.67752 c = 0.07142	0.9989	0.0012	0.0032	0.0012
	T4	a = 0.48561 k 1 = 0.48256 k 2 = 0.53109	0.9991	0.0011	0.0026	0.0012	T5	a = 0.45508 k 1 = 0.41014 k 2 = 0.45329	0.9998	0.0001	0.001	0.0001
	T8	a = 0.47639 k 1 = 0.56369 k 2 = 0.57371	0.9991	0.0026	0.0028	0.0025	T9	a = 0.45571 k 1 = 0.42089 k 2 = 0.44310	0.9992	0.0025	0.0024	0.0014
**Two Term Exponential Model**	T12	a = 0.55683 k 1 = 0.52048 k 2 = 0.61983	0.9976	0.0019	0.0049	0.0024	T13	a = 0.49476 k 1 = 0.49153 k 2 = 0.55900	0.9991	0.0022	0.0028	0.0012
	T16	a = 0.54579 k 1 = 0.63661 k 2 = 0.83403	0.999	0.0005	0.0028	0.0016	T17	a = 0.51851 k 1 = 0.47206 k 2 = 0.54368	0.9918	0.0009	0.0044	0.0024
	T4	k = 0.95204 n = 0.60586	0.9991	0.0027	0.0026	0.0009	T5	k = 0.80175 n = 0.56525	0.9992	0.0025	0.0024	0.0019
	T8	k = 1.26929 n = 0.58674	0.9998	0.0019	0.0014	0.0011	T9	k = 0.82819 n = 0.56794	0.9992	0.0024	0.0024	0.0021
**Page Model**	T12	k = 1.09389 n = 0.61296	0.9991	0.0013	0.0011	0.0009	T13	k = 0.89692 n = 0.56097	0.9991	0.001	0.0028	0.0009
	T16	k = 1.19033 n = 0.57643	0.9996	0.001	0.0016	0.0003	T17	k = 0.93969 n = 0.58209	0.9991	0.003	0.0028	0.0009
	T4	a = 0.89006 k = 0.62321	0.998	0.0144	0.004	0.0023	T5	a = 0.83882 k = 0.55505	0.9988	0..025	0.0039	0.0026
	T8	a = 0.96029 k = 0.66341	0.9982	0.0042	0.004	0.0019	T9	a = 0.84365 k = 0.55611	0.9982	0.019	0.0036	0.0029
**Henderson & Pabis Model**	T12	a = 0.95919 k = 0.63716	0.9887	0.0138	0.0108	0.011	T13	a = 0.86204 k = 0.57304	0.997	0.0246	0.0053	0.0034
	T16	a = 0.97458 k = 0.63442	0.9987	0.0031	0.0034	0.0014	T17	a = 0.88288 k = 0.59799	0.997	0.0172	0.0053	0.0034
	T4	a = 0.67766 b = 0.15900 c = 0.10118 k = 0.70637 g = 0.41594 h = 0.06532	0.9991	0.0049	0.0026	0.001	T5	a = 0.70326 b = 0.16505 c = 0.12958 k = 0.66274 g = 0.43449 h = 0.07144	0.9992	0.003	0.0024	0.0009
	T8	a = 0.66054 b = 0.15568 c = 0.08031 k = 0.73509 g = 0.39540 h = 0.07249	0.9987	0.0042	0.0028	0.0012	T9	a = 0.69245 b = 0.16429 c = 0.12064 k = 0.69714 g = 0.43552 h = 0.05913	0.9982	0.0037	0.0024	0.001
**Modified Henderson & Pabis Model**	T12	a = 0.64672 b = 0.15494 c = 0.09652 k = 0.73903 g = 0.36969 h = 0.07880	0.989	0.0045	0.0034	0.001	T13	a = 0.67921 b = 0.16041 c = 0.12394 k = 0.70599 g = 0.38776 h = 0.06994	0.984	0.0043	0.0031	0.0011
	T16	a = 0.64756 b = 0.15125 c = 0.06532 k = 0.75627 g = 0.40110 h = 0.07360	0.9987	0.0067	0.0033	0.0013	T17	a = 0.67723 b = 0.15808 c = 0.11115 k = 0.70678 g = 0.38778 h = 0.07047	0.9986	0.0055	0.0035	0.0015

Drying Constants and Coefficients (Const. & Coffic.); T2-T5 (WPT); T6-T9 (Soaked); T10-T13 (US 28 kHz); T14-T17 (US 40 kHz).

Among the all tested models, page is the excellent fit models presenting the highest, R^2^ ≥ 0.9993, X ^2^ ≤ 0.0013, RMSE ≤ 0.0018, and RSS ≤ 0.0013, followed by Logarithmic model with the R^2^ ≥ 0.9991, X ^2^ ≤ 0.0021, RMSE ≤ 0.0022, and RSS ≤ 0.0201, Newton model R^2^ ≥ 0.9985, X^2^ ≤ 0.0016, RMSE ≤ 0.0029, and RSS ≤ 0.028, Two term exponential model R^2^ ≥ 0.9981, X^2^ ≤ 0.0019, RMSE ≤ 0.0027, and RSS ≤ 0.0028, Henderson & Pabis Model R^2^ ≥ 0.9977, X ^2^ ≤ 0.0145, RMSE ≤ 0.0038, and RSS ≤ 0.0031 and Modified Henderson & Pabis Model R^2^ ≥ 0.9972, X^2^ ≤ 0.0036, RMSE ≤ 0.0038, and RSS ≤ 0.018 and, at different temperatures, respectively in Table 1.

Page and Logarithmic models showed R^2^ values >0.9991, Newton and Two Term Exponential models had R^2^ values ≥0.9981, whereas Henderson & Pabis Model as well as Modified Henderson & Pabis Model both have R^2^ values of >0.9972 at all temperatures. Thus, the Page model accurately predicted and matched our data, supporting Rudy et al. [59] results on fixed bed cashew drying. Amadeu et al. [60] also demonstrated the Page model for drying germinated faba bean seeds in a thin layer at 50 to 80 °C.

The Page models were also acceptable for predicting the drying kinetic curves for mulatto beans (*Phaseolus vulgaris L.*) with 1.0 m/s drying air speed and for maize seeds at 40 to 60 °C [61]. Furthermore SIQUEIRA et al. [62], used similar drying conditions and obtained logarithmic results. According to Miranda et al. [63], the logarithmic model best predicted convective drying of quinoa at 40–80 °C based on both practical and mathematical data. The logarithmic model's three terms help approximate exponential drying curves mathematically, explaining the excellent match to the data. Tekin and Baslar [64], observed that the Logarithmic model fit better, with R^2^ > 0.9950, for US germinated red peppers subjected to vacuum drying at 45, 55, 65, and 75 °C. However, multiple investigations found that R^2^ is not the sole statistical metric for selecting and evaluating nonlinear mathematical models [[65], [66], [67]]. Thus, X^2^, RMSE, and RSS were also considered.

**Fig. 3:**
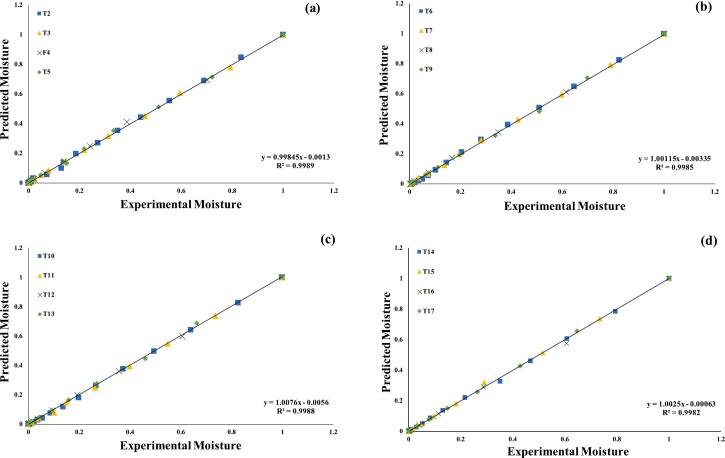
**(a-d).** Validation of the Newton model by comparing predicted vs. experimental data of quinoa grains; WPT (a); soaked (b); US 28 kHz (c); US 40 kHz (d).

**Fig. 4:**
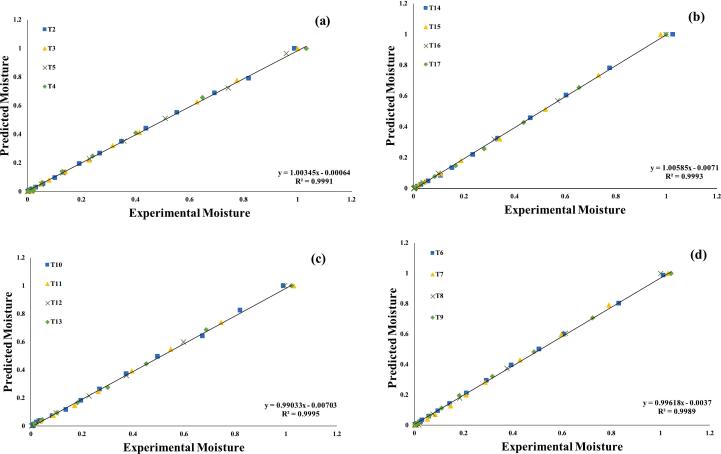
**(a-d).** Validation of the Logarithmic model by comparing predicted vs. experimental data of quinoa grains; WPT (a); soaked (b); US 28 kHz (c); US 40 kHz (d).

**Fig. 5:**
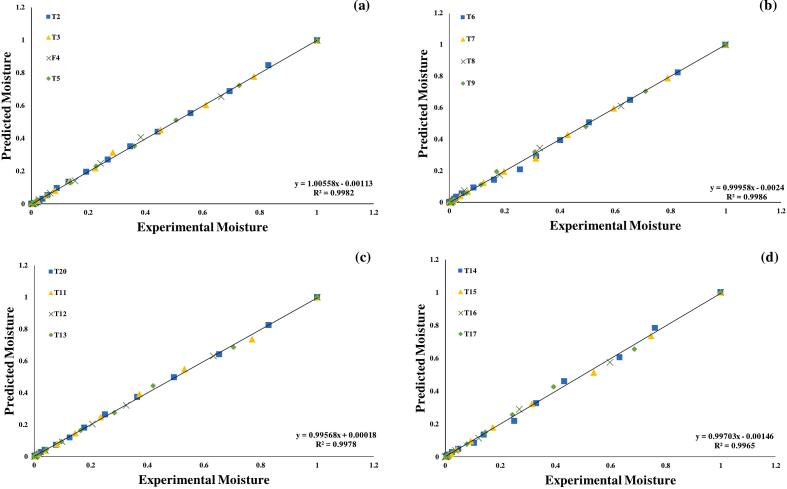
**(a-d).** Validation of the Two term exponential model by comparing predicted vs. experimental data of quinoa grains; WPT (a); soaked (b); US 28 kHz (c); US 40 kHz (d).

**Fig. 6:**
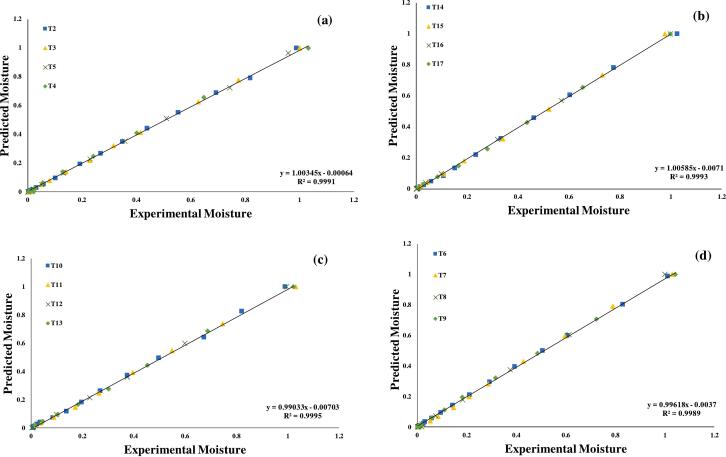
**(a-d).** Validation of the Page model by comparing predicted vs. experimental data of quinoa grains; WPT (a); soaked (b); US 28 kHz (c); US 40 kHz (d).

**Fig. 7:**
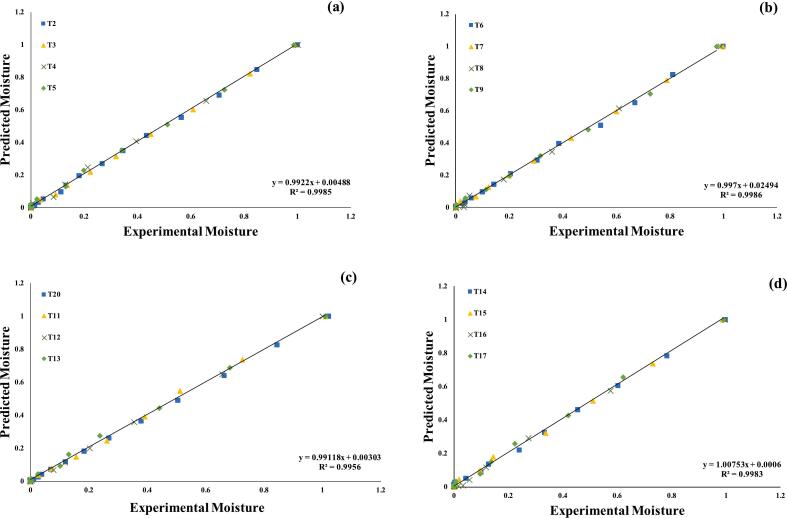
**(a-d).** Validation of the Henderson & Pabis model by comparing predicted vs. experimental data of quinoa grains; WPT (a); soaked (b); US 28 kHz (c); US 40 kHz (d).

**Fig. 8:**
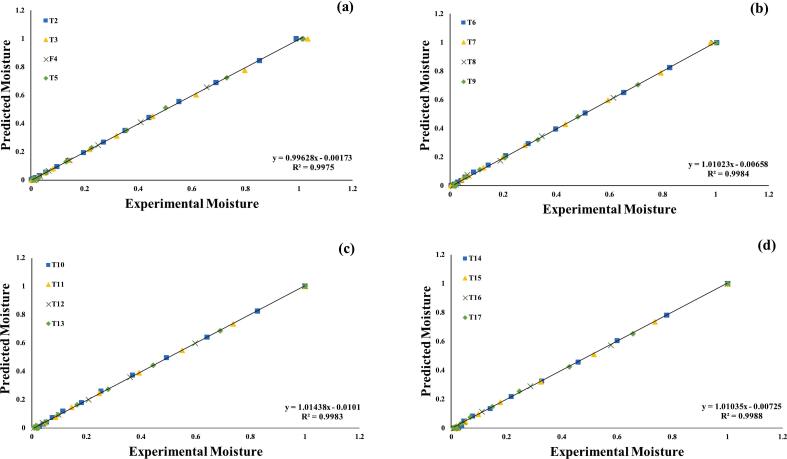
**A-d.** Validation of the Modified Henderson & Pabis model by comparing predicted vs. experimental data of quinoa grains; WPT (a); soaked (b); US 28 kHz (c); US 40 kHz (d).

Furthermore, the seven assessed models were selected for further investigations, using regression analysis, as illustrated in (Fig. 3, Fig. 4, Fig. 5, Fig. 6, Fig. 7, Fig. 8) Newton; Logarithmic; Two-term; Page; Handerson & Pabis and Modified Henderson & Pabis model. All germination groups showed excellent agreement between the predicted and experimental results. The data points gathered around the 45° straight line in Fig. 3, Fig. 4, Fig. 5, Fig. 6, Fig. 7, Fig. 8. That the models can anticipate quinoa grain drying is supported by this trend. Previous investigations have revealed comparable results sample dried with hot air at US 20 kHz, US 40 k Hz, and US 60 kHz [57].

#### 3.1.3 Energy activation (*Ea*)

**Fig. 9:**
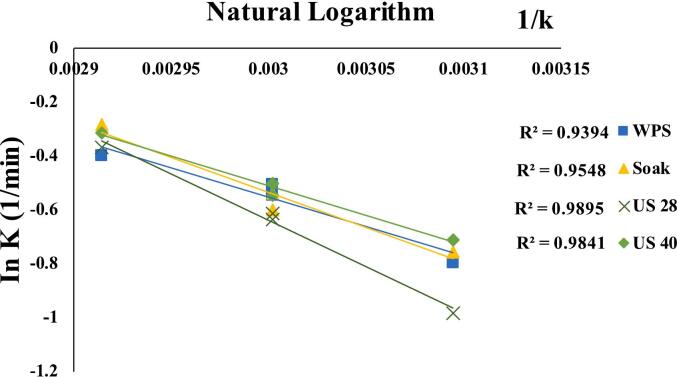
The natural logarithm (ln vs slope 1/k) represents quinoa activation energy (*Ea*).

30480453390000*Ea* is a key component in determining the minimum energy required to initiate a process. *Ea* values in this study varied from 18.25 to 28.41 kJ/mol, and R^2^ values were 0.9394 to 0.9896. Interestingly, the US 40 kHz pre-treated samples consumed 18.21 kJ/mol, the least, followed by the US 28 kHz treatment 28.41 kJ/mol. Another study, Moura et al. [68], dried trapia residues in an air-circulating oven and found that the *Ea* value was 16.61 kJ/mol. Our findings align with Onwude et al. [69], who reported 11.57 to 36.44 kJ/mol in hot air dryers for vegetables. We also validated Ambawat et al. [70] conclusion that untreated samples had a much higher *Ea* value than pre-treated ones. According to Fauzi et al. [71], Food components have *Ea* values ranged from12.7–110 kJ/mol. The linear connection between temperature and *Ea* is seen in Fig. 9. Mass transfer, diffusion, and drying speeds increase with a lower *Ea* value because low energy is required to reach the active state. This tendency is consistent with previous investigations [48]. Activation energy depends on pretreatment, material, and temperature [72].

#### 3.1.4 Effective moisture diffusivity (EMD)

Determining the rate of moisture diffusion is critical for drying agricultural items. Before evaporation gets started, the food products inside moisture must spread to the surface. Based on the data, it implies a decreasing rate drying cycle is typical, as the drying duration is determined by the internal mass transfer rate. Fick's diffusion equation was used for analysing the experimental results and determining *EMD* Fig. 9. In the present study, pre-treated germinated quinoa grains exhibited higher *EMD* values than untreated samples at all drying temperatures. According to Fabani et al. [73], increased drying air temperatures allowed water to be more readily transferred from grain interiors to grain surfaces. Pre-treatments caused structural changes on the quinoa grain, which enhanced moisture loss and a significant rise in *EMD*. Out of all the pre-treatments, the US 40 kHz pre-treatment had the greatest *EMD* at all drying temperatures. Throughout the whole group the *EMD* varied between 3.74 × 10^−9^ m^2^/s and 4.75 × 10^−9^ m^2^/s at 50C. At 70 °C it was 7.09 × 10^−9^ m^2^/s to 8.42 × 10^−9^ m^2^/s, at 60 °C, it ranged from 4.82 × 10–9 to 6.90 × 10 − 9 m^2^/s, while at 50 °C it was 5.85 × 10^−9^ m^2^/s to 6.83 × 10^−9^ m^2^/s. Madamba et al. [74], found the *EMD* values ranging from 10^−9^ to 10^−11^ m^2^/s in agricultural products. Several studies have shown that this might be due to the fact that water molecules become more active and migrate more quickly towards the surface of the matrix as temperatures increase [62]. It is interesting, an increase in temperature caused a decrease in water viscosity and increased the activity of water molecules. These phenomena facilitated diffusion of water molecules in object capillaries and consequently, potentially increased the *EMD* [75]. This type of behaviour was seen in both cowpeas [76] and soybeans [77]. Typically *EMD* values for agricultural goods were found to be between 10^−11^ and 10^−9^ m^2^/s [74].

### 3.2 Effect of pre-treated germination quinoa grains on proximate composition

Table 2 demonstrates the proximal compositions of raw and pre-treated germinated quinoa grains dried at all temperatures. Sprouting moisture content was 72–85 %, while drying moisture contact varied from 3.26 ± 0.02 to 4.87 ± 0.02 (Table 2). Ramos-Pacheco et al., [78], found significant variations in final moisture % after germination (p ≤ 0.05). Regarding protein, germinated have significantly influence the content of protein in all germinated samples. Highest percentage was found in US 40 kHz 22.23 %, followed by US 28 kHz 22.01 % dried at 60 °C. Our results showed that drying germinated quinoa grains at various temperatures increased protein content differently. In WPT and soaked germinated grains, the highest increase were found by sample dried at 50 °C, while in US 28 kHz and US 40 kHz, the highest increase were found at 60 °C. Among germinated groups the highest percentage was found in US 40 kHz 20.12 ± 0.32 % to 22.23 ± 0.03 % followed by US 28 kHz 20.69 ± 0.75 % to 22.01 ± 0.47 %, soaked 19.91 ± 0.32 % to 21.08 ± 0.14 %, WPT 19.6 ± 0.33 % to 21.23 ± 0.13 % than control 16.12 ± 0.03 % (p ≤ 0.05). This range is in agreement with the quinoa protein values reported by Maldonado-Alvarado et al. [79], found 8.0 % to 22.0 % increase in protein content after germination of quinoa grains. According to Fischer et al. [80], quinoa's protein content increased from 14.4 % to 15.5 % after 48 h of germination, and it ranged between 16–19 % [2]. Quinoa samples' proximate analysis variations may be attributed to seed type and growth environment, which may impact protein content [81].

**Table 2:** Proximal compositions of pre-treated germinated quinoa grains.

**Sample code**	**Treatments**	**Moisture content**	**Protein content**	**Fat content**	**Ash content**	**Fiber content**	**Carbohydrate values**	**Energy values**
T1	Control	5.95 ± 0.02a	16.12 ± 0.03^g^	1.87 ± 0.04^a^	2.22 ± 0.02^h^	3.67 ± 0.07^e^	76.7 ± 0.53a	385.79 ± 0.49a
T2	WPT	4.34 ± 0.02f	21.23 ± 0.13^bc^	1.57 ± 0.05^cdef^	2.66 ± 0.04^a^	4.31 ± 0.02^cd^	70.28 ± 0.07de	379.68 ± 0.43bcde
T3		4.23 ± 0.02 g	20.15 ± 0.11^def^	1.27 ± 0.03^hi^	2.56 ± 0.01^bcd^	4.41 ± 0.02^c^	71.62 ± 0.13 fg	378.5 ± 0.29 fg
T4		4.36 ± 0.02f	19.6 ± 0.33^f^	1.37 ± 0.03^ghi^	2.42 ± 0.03^g^	4.21 ± 0.02^d^	72.41 ± 0.29b	380.33 ± 0.19bc
T5		4.62 ± 0.02d	20.47 ± 0.29^bcdef^	1.35 ± 0.02^ghi^	2.52 ± 0.02^def^	4.69 ± 0.03^ab^	70.97 ± 0.23 g	377.91 ± 0.06 g
T6	Soaking	4.87 ± 0.02b	19.91 ± 0.32^ef^	1.37 ± 0.03^ghi^	2.61 ± 0.03^abcd^	4.19 ± 0.16^d^	71.93 ± 0.25bc	379.65 ± 0.79bcde
T7		3.45 ± 0.02i	20.19 ± 0.17^def^	1.39 ± 0.05^fgh^	2.61 ± 0.03^abc^	4.24 ± 0.03^cd^	71.56 ± 0.17bc	379.51 ± 0.43cdef
T8		3.87 ± 0.02 h	20.36 ± 0.28^cdef^	1.54 ± 0.05^cde^	2.55 ± 0.035^bcd^	4.65 ± 0.02^b^	70.91 ± 0.33cde	378.91 ± 0.45ef
T9		4.31 ± 0.02f	21.08 ± 0.14^bcd^	1.57 ± 0.05^cd^	2.59 ± 0.041^abcd^	4.33 ± 0.01^cd^	70.44 ± 0.25de	380.18 ± 0.09bcd
T10	US 28	4.25 ± 0.02 g	20.73 ± 0.51^bcde^	1.41 ± 0.06^efgh^	2.62 ± 0.02^ab^	4.36 ± 0.04^cd^	70.88 ± 0.58cde	379.11 ± 0.27def
T11		3.87 ± 0.02 h	22.01 ± 0.47^a^	1.72 ± 0.04^b^	2.65 ± 0.013^a^	4.75 ± 0.03^ab^	68.17 ± 0.54ef	379.02 ± 0.20ef
T12		3.26 ± 0.02j	20.69 ± 0.75^bcde^	1.42 ± 0.04^efg^	2.45 ± 0.026^fg^	4.23 ± 0.13^cd^	71.21 ± 0.88cde	380.35 ± 0.34bc
T13		4.32 ± 0.01 g	21.16 ± 0.14^bcd^	1.25 ± 0.04^i^	2.46 ± 0.01^efg^	4.23 ± 0.04^cd^	70.9 ± 0.16cde	379.47 ± 0.25cdef
T14	US40	4.56 ± 0.02e	20.12 ± 0.32^def^	1.65 ± 0.08^bc^	2.55 ± 0.03^bcd^	4.34 ± 0.04^cd^	71.34 ± 0.25 cd	380.67 ± 0.65b
T15		3.49 ± 0.03i	22.23 ± 0.03^a^	1.45 ± 0.03^def^	2.55 ± 0.02^bcd^	4.82 ± 0.03^a^	68.16 ± 0.05 g	377.45 ± 0.26 g
T16		3.88 ± 0.02 h	20.91 ± 0.40^bcde^	1.37 ± 0.03^ghi^	2.52 ± 0.04^def^	4.28 ± 0.07^cd^	70.92 ± 0.31cde	379.61 ± 0.38bcde
T17		4.71 ± 0.02c	21.46 ± 0.25^b^	1.47 ± 0.07^def^	2.53 ± 0.03^cde^	4.31 ± 0.01^cd^	70.23 ± 0.16e	379.95 ± 0.21bcde

Data are pretested in (means ± standard deviation) of three replicates (n = 3); proximate data expressed %, Energy kcal/100 g on dry matter basis; (a-i), shows statistical differences (p ≤ 0.05).

Fat content considerably reduced in treatment groups: control: 1.87 ± 0.04 %, WPT: 1.27 ± 0.03 % to 1.57 ± 0.05 %, soaking: 1.37 ± 0.03 % to 1.57 ± 0.05 %, US 28KHz: 1.25 ± 0.04 % to 1.72 ± 0.04 %, and US 40 kHz: 1.37 ± 0.03 % to 1.65 ± 0.08 % (Table 2). We discovered similar findings to Ramos-Pacheco et al. [78], who showed significant ash increases during quinoa grain germination in white, red, and black verities. Maldonado-Alvarado et al., [79], found that germination increased ash by 9–18 % over soaking (1.78–3.88 %). Fibre content increased with US 40KHz dried sample at 60 °C the highest (31.39 %), followed by US 28 kHz (29.71 %) (p ≤ 0.001). Higher cavitation effects may explain why US 40 kHz at 60 °C increased fibre content by the most (31.39 %). US 28 kHz ultrasound generated more fibre, but less than 40 kHz (29.71 %), probably because to cavitation intensity fluctuations. Our results correlate with Deore et al. [82], who found 51.13 % and 45.11 % increase in fiber in US 10 and 20 kHz pre-treated sample than control. Given the increased interest in plant-based ingredients, quinoa germination may increase its fibre content for food preparation.

Drying quinoa grains substantially reduced total carbohydrate content across treatments. The greatest drop was seen in US 40 kHz at 60 °C (68.16 ± 0.05 %), followed by US 28 kHz (68.17 ± 0.54) and WPT at 50 °C (70.28 ± 0.07). This is due to the metabolic activity of the seeds. Sprouting rice, sorghum, millet, buckwheat, and amaranth decreased carbohydrate content by 5–51 % [83]. High-protein, low-carbohydrate foods are healthy since they don't boost plasma glucose. Quinoa control sample had the greatest energy (385.79 ± 0.49 kcal), whereas germinated samples varied from 380.67 ± 0.65 to 377.45 ± 0.26 kcal. Similarly, Pathan et al. [7] found energy in Quinoa grains 331–381 kcal, Padmashree et al. [84] found germinated sample has 336.38 kcal.

### 3.3 Total flavonoids and phenolic content (TFC & TPC)

Table 3 shows the TFC and TPC values of both the control and pre-treated assisted germinated grains that were dried at various temperatures. A significant increase of TPC as TFC values throughout the germination was also observed. In this study, the TFC content in control was 10.59 ± 0.35 mg QE/100 g, while in WPT germinated sample at all drying temperatures was from 12.9 ± 0.21 to13.69 ± 0.43 mg QE/100 g increased by (11.8 to 29.3 %), Soaking 10.87 ± 0.38 to 14.86 ± 0.72 mg QE/100 g increased by (2.6 to 40 %), US 28KHz 12.22 ± 0.62 to 16.94 ± 0.51 mg QE/100 g increased by (15.4 to 59.9 %), and US 40KHz 11.45 ± 0.74 to16.77 ± 0.52 mg QE/100 g increased by (8.1 to 58.2 %) than control samples (Table 3). The results were comparable to those indicated by Carciochi et al. [85], who showed a range of TPC in pre-treated quinoa grains 11.06 ± 0.42 to 17.65 ± 0.45 mg QE/100, increased by 59.5 % than the control after germination (p < 0.001).

**Table 3:** Impact of drying temperatures on pretreated assisted germinated quinoa grains; TFC, TPC and antioxidant properties.

**Sample code**	**Treatments**	**TFC mg (mgQE/100 g)**	**TPC mg (mgGAE/100 g)**	**DPPH** **(%)**	**FRAP uM (μmol TE/g)**	**ABTS uM (μmol TE/g)**	**Reducing power (mgAAE/g)**	**Metal Chelating (%)**
T1	Control	10.59 ± 0.35^g^	32.25 ± 0.27^k^	42.94 ± 0.03^i^	6.98 ± 0.04^g^	10.27 ± 0.025^i^	2.19 ± 0.04^de^	21.42 ± 0.53^h^
T2	WPT	13.69 ± 0.43^bc^	54.47 ± 0.83^h^	52.54 ± 0.17^bcd^	9.02 ± 0.07^abcd^	13.46 ± 0.013^d^	2.47 ± 0.01^c^	34.78 ± 0.47^c^
T3		12.9 ± 0.21^cde^	59.86 ± 0.45^d^	50.58 ± 0.32^efg^	8.09 ± 0.11^defg^	14.26 ± 0.14^c^	2.25 ± 0.08^de^	38.9 ± 0.43^b^
T4		13.46 ± 0.36^cd^	55.15 ± 0.43^gh^	50.25 ± 0.16^fg^	7.39 ± 0.04^fg^	12.63 ± 0.06^f^	1.97 ± 0.03^f^	25.16 ± 0.40^f^
T5		11.84 ± 0.41^efg^	46.48 ± 0.27^j^	49.79 ± 0.02^g^	8.79 ± 0.03^bcde^	12.17 ± 0.01^g^	2.13 ± 0.01^e^	25.55 ± 0.52^f^
T6	Soaking	14.86 ± 0.72^b^	52.88 ± 0.63^i^	48.33 ± 0.06^h^	9.23 ± 0.06^abcd^	14.44 ± 0.25^bc^	2.44 ± 0.04^c^	39.58 ± 0.42^b^
T7		14.2 ± 0.24^bc^	59.84 ± 0.73^d^	51.84 ± 0.24^cde^	9.08 ± 0.14^abcd^	13.7 ± 0.08^d^	2.19 ± 0.06^de^	27.38 ± 0.47^e^
T8		10.87 ± 0.38^g^	62.37 ± 0.61^bc^	50.9 ± 0.05^efg^	7.67 ± 0.07^efg^	11.96 ± 0.14^gh^	1.99 ± 0.01^f^	19.58 ± 0.59^i^
T9		13.08 ± 0.36^cde^	58.32 ± 0.27^e^	52.63 ± 0.28^bcd^	8.52 ± 0.13^cdef^	12.96 ± 0.26^e^	2.87 ± 0.04^a^	27.92 ± 0.31^de^
T10	US 28	14.03 ± 0.47^bc^	56.24 ± 0.41^fg^	52.63 ± 0.07^bcd^	10.37 ± 0.11^a^	14.61 ± 0.01^b^	2.64 ± 0.08^b^	40.02 ± 0.73^b^
T11		16.94 ± 0.51^a^	61.77 ± 0.55^c^	53.37 ± 0.09^b^	10.02 ± 0.04^ab^	14.59 ± 0.61^b^	2.91 ± 0.09^a^	41.67 ± 0.21^a^
T12		12.22 ± 0.62^def^	57.02 ± 0.29^f^	51.43 ± 0.17^def^	9.68 ± 0.09^abc^	12.9 ± 0.07^e^	2.14 ± 0.12^e^	23.31 ± 0.46^g^
T13		13.01 ± 0.35^cde^	58.32 ± 0.17^e^	52.47 ± 0.03^bcd^	9.09 ± 0.15^abcd^	13.49 ± 0.15^d^	2.46 ± 0.04^c^	28.91 ± 0.46^d^
T14	US40	13.4 ± 0.46^cd^	63.41 ± 0.87^ab^	53.21 ± 0.21^bc^	10.33 ± 0.13^a^	14.29 ± 0.24^c^	2.81 ± 0.06^a^	42.13 ± 0.42^a^
T15		16.77 ± 0.52^a^	64.43 ± 0.31^a^	54.88 ± 0.17^a^	10.14 ± 0.05^ab^	15.34 ± 0.08^a^	2.92 ± 0.05^a^	42.13 ± 0.28^a^
T16		11.45 ± 0.74^fg^	56.84 ± 0.24^f^	50.43 ± 0.22 f^g^	9.55 ± 0.08^abc^	12.65 ± 0.05^f^	2.16 ± 0.14^e^	28.51 ± 0.54^de^
T17		14.26 ± 0.73^bc^	59.88 ± 0.58^d^	48.2 ± 0.31^h^	10 ± 0.17^ab^	11.9 ± 0.14^h^	2.31 ± 0.07^d^	28.65 ± 0.48^de^

Data are pretested in (means ± standard deviation) of three replicates (n = 3); (a-k), shows statistical differences (p ≤ 0.05); Without Pre-treatment (WPT); Ultrasound 28 kHz (US 28); Ultrasound 40 kHz (US 40); DPPH, 2,2-diphenyl-1-picrylhydrazyl radical; FRAP, Ferric reducing antioxidant power; ABTS, 2,2-azinobis(3-ethylbenzothiazoline-6-sulfonic acid).

TPC in control was 32.25 ± 0.27 mg GAE/100 g increased by 46.48 ± 0.27 to 59.86 ± 0.45 mg GAE/100 in WPT, 52.88 ± 0.63 to 62.37 ± 0.61 mg GAE/100 in soaking, 56.24 ± 0.41 to 61.77 ± 0.55 mg GAE/100 in US 28 kHz, and 56.84 ± 0.24 to 64.43 ± 0.31 mg GAE/100 in US40 kHz(p < 0.001). Guardianelli et al. [8] determined the TPC of white quinoa 94.3 ± 12.4 mg GAE/100 g of flour, whereas Bhinder et al. [86] found it to be 3.61 ± 0.46 mg GAE/100 (p < 0.001). Germinated quinoa grains increased TPC by 31.7, 43.8, and 29.3 % [5]. Interestingly, US 28 kHz dried at 50 °C increased TFC, whereas US 40 kHz dried at 60 °C increased TPC the most. Ultrasonic cavitation in US pre-treatments increases flavonoid and total phenolic acid content by mechanically stressing materials and releasing more flavonoids [87]. TFC and TPC in Andean grains vary according on the cultivar, growth stage, growing area, and season [88].

### 3.4 Antioxidant Activates

Quinoa grains showed higher antioxidant activity and significant (P ≤ 0.001) after pre-treated germination followed by drying at various temperatures (Table 3). DPPH scavenging was (49.79 to 52.54 %) without pre-treatment, 48.33 to 52.63 % soaking, 51.43 to 53.37 % US28 kHz, and 50.43 to 53.21 % US 40 kHz, whereas 42.94 % in control. Similar to our findings, Huang et al. [89], found, DPPH scavenging activity increased from 56.19 to 70.49 % mung bean seed after pre-treated sample followed by germination. While Carciochi, et al. [85], reported 21.8 % and 65.02 % DDPH following quinoa germination.

Ferric ion reducing antioxidant power (FRAP) increased with all treatments, especially drying at 50 °C. Without pre-treatments, FRAP increased by 9.02 ± 0.07 μmol TE/g, followed by Soaked (9.23 ± 0.06), Ultrasound 28KHz (10.37 ± 0.11), and Ultrasound 40Khz (10.33 ± 0.13) (p < 0.001). The lowest increase in FRAP value was observed in samples dried at 70 °C, with values of 7.39 ± 0.04 μmol TE/g for WPT and 7.67 ± 0.01 μmol TE/g for soaked samples. Kittibunchakul et al. [90] demonstrated that low-temperature processing significantly enhanced FRAP values in seeds (p ≤ 0.05). Consistent with these findings, Siriparu et al. [91] reported a 36.6 % increase in mung beans, while Namerata et al. [92] observed a 48.68 % increase in FRAP after germination of quinoa grains followed by drying at low-temperature. These findings are consistent with our results, which showed a FRAP increase ranging from 9.58 % to 48.04 %.

Quinoa's antioxidant activity ABTS increased significantly (p < 0.001). Control sample had ABTS value of 10.27 ± 0.025 µM TE/g. Germination treatments led to higher values under all conditions, including WPT (12.17 ± 0.01 to 14.26 ± 0.14 µM TE/g), soaking (11.96 ± 0.14 to 14.44 ± 0.25), US 28 kHz (from 12.90 ± 0.07 to 14.61 ± 0.01), and US 40 kHz (from 11.90 ± 0.14 to 15.34 ± 0.08 µM TE/g). Table 3 shows that the US 40 kHz treatment sample dried at 60 °C showed the highest values of ABTS 15.34 ± 0.08 µM TE/g (p < 0.001). ABTS levels decreased significantly in samples dried at higher or mixed temperatures, but remained higher than the control. Repo-Carrasco-Valencia & Serna [6] found similar results, with ABTS values ranging from 9.40-14.74 µmol TE/g DW. Furthermore, Li et al. [93], showed lower ABTS values (4.08 ± 3.98 to 8.61 ± 1.67 µmol TE/g) in extracts from 13 quinoa types, whereas our findings were much higher. According to Wang et al. [94], pre-treated quinoa grains had 16.6 %, 11.1 %, and 15.0 % higher ABTS scavenging capability than untreated samples. Hou et al. [95], found that germination increased ABTS free radical scavenging by 69.97 %. These data support germinated quinoa as an antioxidant source, particularly when pre-treated.

Drying at moderate temperatures considerably increased antioxidant reducing power, with a significant increase seen at Ultrasound 40 kHz (2.92 ± 0.05 µmol AAE/g), followed by Ultrasound 28KHz (2.91 ± 0.09 µmol AAE/g), compared to the control (2.19 ± 0.04 µmol AAE/g) (Table 3). Drying at 70 °C considerably reduced reducing power, with significant decreases in WPT (1.97 ± 0.03 µmol AAE/g), soaked (1.99 ± 0.01 µmol AAE/g), US 28 kHz (2.14 ± 0.12), and US 40 kHz (2.16 ± 0.14) (p ≤ 0.001). Złotek et al. [96], found a considerable decrease in reducing power following germination and high-temperature drying. Similarly, sample dried at 70 °C greatly lowered metal chelating, although it was still higher than control. Our results support the findings of An et al. [97], food items should be dried at 60 °C for a shorter time to preserve their antioxidant capabilities. Germinated amaranth flours have 54.3 % more antioxidant activity than un-germinated [98]. Barley, brown rice, oats, and quinoa exhibit antioxidant activity that rise by 7.5–190 % following germination (15–28 °C, 2–5 days). Furthermore, Altıkardeş et al. [34] discovered that quinoa's antioxidant capacity was considerably increased by US pre-treatment assisted germination 64 % and soaking by 2 % compared to control. Overall, our findings showed that US 40 assisted germination sample dried at 60 °C increased the antioxidant activities (metal chelating, DPPH, FRAP, ABTS, and reducing power). Previously, An et al. [97] reported that food items need to be dried at a lower temperature (60 °C) for a shorter duration in order to preserve their antioxidant qualities. Ren et al. [30] demonstrated that the higher antioxidant activity observed may improve antioxidant element extraction efficiency after drying. This may explain the increased in the antioxidants activities observed in germinated samples dried at 60 °C.

### 3.5 Antioxidant potency composite index (APCI)

The various antioxidant assays exhibit variations in principles, mechanisms and experimental conditions due to which antioxidants exhibit different antioxidant potency among five studied antioxidant studies (Table 4**)**. An overall APCI was calculated for each variety using the method described by Seeram et al. [99]. Overall, Table 8 shows the raw sample has the lowest APCI (67.64 %). Among germinated samples, US 40 kHz sample dried at 60 °C showed quinoa grains had the highest APCI (98.78 %) and the lowest in the WPT group (74.67 %). In the group comparison, US 40 kHz germination had the highest APCI (90.09 %), followed by US 28 kHz (89.34 %), Soaking (82.38 %), and WPT (81.53 %). In WPT, the sample dried at 50 °C (87.52 %) had the highest APCI and the lowest at 70 °C (74.64 %). In soaked assisted germination, the sample dried at 50 °C (89.74 %) had the highest APCI while lowest at 70 °C (75.84 %). In US 28 kHz germinated samples dried at 60 °C showed the highest APC (96.78 %) and the lowest at combined temperatures (82.15 %). We found that drying at 60 °C following ultrasound treatment at 40 kHz yielded the greatest APCI (98.78 %), suggesting that this combination of ultrasonic frequency and temperature preserved or enhanced antioxidants. Overall, germinating samples dried at different temperatures exhibited greater APCI than raw samples. According to the literature search, no research has examined the APCI of both control and pre-treated (WPT, Soaked, US 28 kHz & US 40 kHz) quinoa grains before germination, dried at various temperatures as well as combined temperatures.

**Table 4:** Antioxidant potency composite index of studied antioxidant activities.

**Sample code**	**Treatments**	**APCI**	**DPPH Index**	**FRAP Index**	**ABTS Index**	**R.P Index**	**MCA Index**
T1	Control	67.64	78.24	67.3	66.91	74.9	50.84
T2	WPT	87.52	95.73	86.98	87.74	84.63	82.54
T3		86.45	92.16	77.96	92.92	76.87	92.33
T4		74.67	91.56	71.28	82.31	67.58	60.64
T5		77.46	90.72	84.75	79.31	72.81	59.7
T6	Soaking	89.74	88.06	88.96	94.11	83.61	93.95
T7		82.23	94.46	87.55	89.26	74.9	64.97
T8		75.84	92.74	73.97	77.94	68.28	66.26
T9		81.70	95.89	82.17	84.49	99.47	46.48
T10	US 28 kHz	86.82	95.9	100	95.25	74.33	68.62
T11		96.78	97.24	96.6	95.1	100	94.98
T12		92.06	93.71	93.35	84.09	90.27	98.89
T13		82.15	95.6	87.62	87.91	84.31	55.32
T14	US40 kHz	97.55	96.95	99.57	93.11	98.13	99.98
T15		98.78	100	97.73	100	96.16	100
T16		81.80	91.88	92.07	82.47	74.9	67.67
T17		81.78	87.82	96.46	77.53	79.08	68.24

### 3.6 Effect of pre-treated germination quinoa grains on colour changes

Color changes of pre-treated germinated quinoa grains dried at various temperatures are presented in Table 5. Pretreatments decreased the lightness L*, while increasing a* and b* of quinoa flour. For example control sample L*, a* and b* values were 66.81, 8.45 and 21.92, respectively. The L* decreases in the WPT it was 54.19 to 55.69, Soaked 53.68 to 59.05, US 28KHz 43.44 to 56.25 and US40KHz 48.65 to 53.44. Similar to our results Altıkardeş et al. [34], measured higher L* values (85.36 ± 0.27) in un-germinated quinoa and found a reduction in germinated sample (7.6 %) and this reduction was associated with retreatments prior to germination. Olojede et al. [100], found un-germinated buckwheat and quinoa samples had L* values of 86.90 ± 0.34 and 85.36 ± 0.27, respectively. In germinated quinoa, Ultrasound 40 dried at 70 °C (12.56) had the highest a* increase, whereas Ultrasound 40 dried at 50 °C (7.33) had the lowest. We discovered that ultrasonic treatment substantially increased b* of quinoa flour more than a* (p ≤ 0.05). Giancaterino et al. [101], found positive Chroma a* and b* and decreased L*. Total colour changes (ΔE) indicate that US40 kHz pre-treatment decrease quinoa.

**Table 5:** Colour changes of pre-treated germinated quinoa grains after drying.

**Sample code**	**Treatments**	L*	a*	b*	**Hue Angle (H**°**)**	ΔE*	Chroma(C*)	**BI**
T1	Control	66.81 ± 1.54^a^	8.45 ± 0.24^cde^	21.92 ± 0.40^h^	21.11 ± 0.03b	0 ± 0	23.5 ± 0.06^k^	12.09 ± 0.176^f^
T2	WPT	54.77 ± 0.71^c^	8.74 ± 0.29^bcde^	28.57 ± 0.91^bcde^	18.32 ± 0.02^e^	8.67 ± 0.05^i^	22.01 ± 0.10*^m^*	16.35 ± 0.64^de^
T3		55.53 ± 0.35^c^	10.98 ± 0.463^ab^	31.01 ± 0.51^a^	18.46 ± 0.09^d^	11.26 ± 0.05^e^	28.68 ± 0.03^g^	19.33 ± 1.48^bc^
T4		54.19 ± 0.99^cd^	10.72 ± 1.34^abc^	28.53 ± 0.81^bcde^	12.55 ± 0.01*^m^*	6.58 ± 0.09^j^	28.22 ± 0.05^h^	18.99 ± 0.81^bc^
T5		55.69 ± 1.18^c^	9.68 ± 0.31^bcde^	30.52 ± 0.71^ab^	16.04 ± 0.04^h^	3.94 ± 0.04*^m^*	20.89 ± 0.09^o^	17.6 ± 1.38^cd^
T6	Soaking	56.07 ± 1.05^c^	7.94 ± 0.90^de^	26.17 ± 0.65^fg^	14.64 ± 0.00*^k^*	14.22 ± 0.01^d^	35.37 ± 0.01^b^	14.55 ± 0.31^e^
T7		59.05 ± 1.34^b^	7.68 ± 1.06^e^	29.01 ± 0.77^abcd^	16.8 ± 0.02^g^	1.95 ± 0.05^p^	23.35 ± 0.02^l^	14.02 ± 0.74^ef^
T8		53.75 ± 1.06^cd^	10.56 ± 1.20^abc^	27.08 ± 0.81^defg^	15.86 ± 0.02^i^	9.75 ± 0.08^h^	29.29 ± 0.05^f^	18.66 ± 0.79^bc^
T9		53.68 ± 0.87^cd^	10.23 ± 0.81^abcd^	29.79 ± 0.47^abc^	15.43 ± 0.04^j^	2.29 ± 0.04^o^	23.58 ± 0.02^j^	18.81 ± 0.85^bc^
T10	US 28	50.05 ± 0.95^ef^	7.71 ± 0.76^e^	26.23 ± 1.15^fg^	26.19 ± 0.06^a^	15.7 ± 0.00^a^	32.94 ± 0.08^e^	16 ± 1.214^de^
T11		50.14 ± 1.24^ef^	8.72 ± 0.63^bcde^	25.06 ± 0.61^g^	12.9 ± 0.05^l^	4.13 ± 0.08^b^	36.92 ± 0.09^a^	16.01 ± 0.17^de^
T12		51.63 ± 0.99^de^	10.71 ± 0.88^abc^	22.39 ± 0.68^h^	18.25 ± 0.04^e^	3.43 ± 0.06^c^	34.4 ± 0.04^c^	19.01 ± 0.49^bc^
T13		43.44 ± 0.72^f^	7.67 ± 0.87^e^	22.45 ± 1.22^h^	10.88 ± 0.10*^n^*	10.5 ± 0.03^g^	26.64 ± 0.04^i^	17.46 ± 0.93^cd^
T14	US40	51.60 ± 0.81^de^	7.33 ± 1.01^e^	26.33 ± 0.90^fg^	17.23 ± 0.05^f^	4.31 ± 0.09^l^	20.47 ± 0.05^p^	14.84 ± 0.88^e^
T15		51.494 ± 0.14^de^	8.74 ± 1.12^bcde^	26.6 ± 0.50^efg^	18.95 ± 0.07^c^	3.71 ± 0.03^f^	33.21 ± 0.06^d^	17.64 ± 0.76^cd^
T16		48.65 ± 1.53^g^	12.56 ± 1.26^a^	27.89 ± 1.05^cdef^	19.06 ± 0.05^c^	3.73 ± 0.05*^n^*	20 ± 0.03^q^	23.51 ± 0.71^a^
T17		51.59 ± 0.51^de^	10.63 ± 0.35^abc^	30.26 ± 0.78^ab^	16.76 ± 0.05^g^	4.75 ± 0.06^k^	21.22 ± 0.00*^n^*	20.18 ± 0.80^b^

Data are pretested in (means ± standard deviation) of three replicates (n = 3); (a-p), shows statistical differences (p ≤ 0.05).

color changes during germination. Chemical processes involving pigments, such as the Millard reaction and melanoidin production after drying, may explain ΔE variation across different temperatures. According to earlier studies, higher-quality dried goods had lower ΔE values [39]. The quantitative feature of colorfulness, known as Chroma (C*), measures the degree of hue difference compared to a grey color of the same light. The results showed that the changes in C* were greater in the ultrasound pre-treated samples. These changes might be related to the influence of ultrasound cavitation, revealing the yellow pigment attached to the intracellular structure of the samples [57]. Similarly, Costa et al. [102] demonstrated that the sonicated pineapple juice had a higher C* value than non-sonicated juice samples (p ≤ 0.05).

Browning index (BI) showed a little decrease across all treatment groups when dried at various temperatures, although BI increased significantly across all groups, particularly at 70 °C Celsius (p ≤ 0.05). Maillard processes, caramelization and phenolic component oxidation are temperature-sensitive and much more active at 70 °C, which is why BI increases at that temperature. Additionally, a same pattern was found, in the studies conducted by Kewuyemi et al. [103] in whole grains biscuits, Soontharapirakkul et al. [104] in germinated mushrooms, and Tian et al. [105], in germinated soybeans.

### 3.7 Polyphenol oxidase (PPO) & peroxidase (POD)

**Fig. 10:**
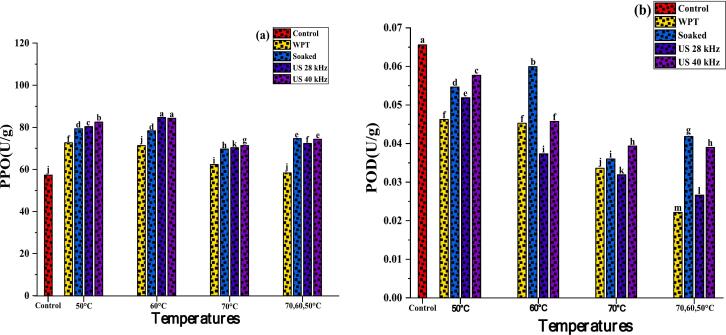
**(a-b);** Effects of pre-treatments on germinated quinoa grains followed by dried in hot air dryer at various temperatures. Polyphenol Oxidase (PPO) (a); Peroxidase (POD) (b). Data were taken in replicate, (a-m). shows statistical differences (p ≤ 0.001).

PPO activity is the primary enzyme that oxidises phenolic compounds into Quinone molecules [106]. Fig. 10**a** showed that the PPO activity of germinated quinoa grains increased in all samples, notably ultrasonic pre-treated groups. Among all germinated samples, higher PPO levels were found in sample dried at lower temperatures than those dried at 70 °C (p ≤ 0.001). These data indicate that polyphenol oxidase enzyme activity increases with germination, dried at 50 °C and 60 °C, and subsequently drops at 70 °C. It is previously stated that drying at moderate temperatures retains enzyme structure and activity [107]. This explains why PPO activity is strong at these temperatures. Our findings match Iqbal et al. [108], who found PPO activity of 91.3 %, 43.3 %, 7.8 %, and 29.3 % in four quinoa genotypes. The activity of PPO in dried quinoa grains exhibited significant variation, highlighting the influence of acoustic treatment followed by drying at different temperatures.

As seen in Fig. 10**b**, germinated quinoa grains may have greater peroxidase activity in control sample. Dried quinoa grains have lower POD activity because drying temperature affects peroxidase activity after germination. Peroxidase activity may be preserved at 50 °C (1.63 to 1.67 U/g) while at 70 °C severely degraded enzyme performance (0.62 to 0.82 U/g) (p ≤ 0.001). Other researchers have shown that high heat lowers PPD levels [109]. Rashid et al. [57] reported similar findings in their study on the effects of ultrasound pre-treatment followed by hot air drying at 60 °C, 70 °C, and 80 °C on sweet potatoes. The authors concluded that drying at 60 °C was optimal for POD activity. In contrast, a significant reduction in POD activity (71.86 %) was observed when samples were dried at 80 °C. Furthermore, Magangana et al. [110] examined the influence of pre-treatments on PPD content of fruits and found that at 60 °C, PPD concentration ranged from 5.25 ± 0.00 to 4.50 ± 0.00, decreasing as temperature increased up to 80 °C. 2.25 ± 0.00 to 1.5 ± 0.00; at 100 °C, 1.13 ± 0.38 to 1.50 ± 0.00 (P ≤ 0.05).

### 3.8 Effect of pre-treated germination quinoa grains on phytic acid and tannins

**Fig. 11:**
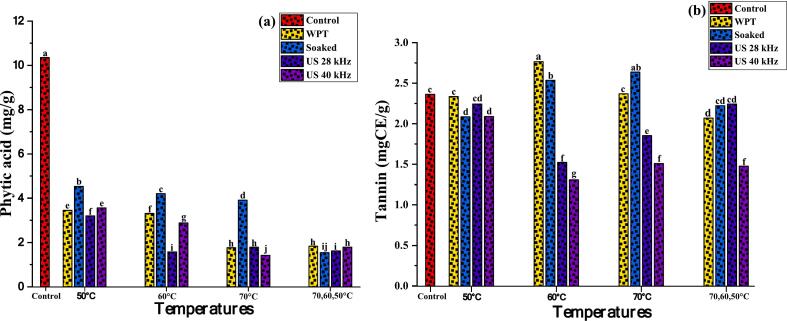
**(a-b);** Effects of pre-treatments on germinated quinoa grains followed by dried in hot air dryer at various temperatures. Phytic acid (a); Tannins (b). Data were taken in replicate, (a-m). shows statistical differences (p ≤ 0.05).

Phytic acid and tannin levels in raw and pre-treated germinated quinoa are presented in (Fig. 11**a-b**). Control sample exhibited the greatest phytic acid content (10.3 mg/g) compared to germinated samples WPT (1.83–3.45 mg/g), soaking (1.55–4.53 mg/g), US 28 kHz (1.62–3.23 mg/g), and US 40 kHz (1.80–3.57 mg/g). Significant reduction in phytic acid content were found among germinated sample at all drying temperatures, WPT shows (66.66 %, 68.01 %, 82.99 %, 82.31 %), Soaked (56.23 %, 59.42 %, 62.22 %, 85.12 %), US 28 (69.08 %, 84.83 %, 82.80 %, 84.34 %) and US (65.60 %, 72.17 %, 86.28 %, and 82.70 %) than control (p ≤ 0.05) (Fig. 11**a**). Our results highlights that ultrasound pre-treated germination followed by drying at 60 °C reduce phytic acid in all treated groups. Moderate heat at 60 °C may maintain or slightly increase phytase enzyme activity, which degrades remaining phytic acid during drying. Our findings align with Altıkardeş etal. [34], who showed that ultrasonic (85.5 %) and soaking (80.6 %) treatments considerably reduced phytic acid levels in germinated quinoa compared to the original control (p ≤ 0.05). Similarly, Kumari et al. [111], showed that phytic acid concentration reduced (55.5 %) after germination of buckwheat, while Bhinder et al. [86], reported that black and white quinoa phytic acid content decreased (59–64 %) after 96 h.

Fig. 11**b** shows that the tannin level in germinated samples, WPT ranged from 2.07 to 2.77, soaked from 2.09 to 2.54, and ultrasound from 1.52 to 2.24 mgCE/g (p ≤ 0.05). We found that tannin concentration in control sample was 2.36 mg TE/g, but it reduced in US 28 kHz and US4 kHz, notably at 60 °C (31.48 %) and (41.60 %). Ultrasound converts hydrolysable tannic acid into gallic acid, lowering sample tannin concentration. In addition to reducing overall tannin concentration, it causes the condensed tannin to leach out of the sample [112]. Thakur et al. [18] observed that pre-treatment for grain germination reduced tannin concentration by 32.31 %. Furthermore, Siwatch et al. [113] and Sindhu et al. [114] also discovered comparable results; after 48 h of germination, there was a significant drop in tannin concentration, which was explained by tannin leaching during pre-treatments assisted germination of amaranth and quinoa grains. The variations in the results could be attributed to differences in the duration of germination, pre-treatment method, temperature conditions applied, and the types of grains utilized in the studies.

### 3.9 Correlation heat map and principal component analysis (PCA)

**Fig. 12:**
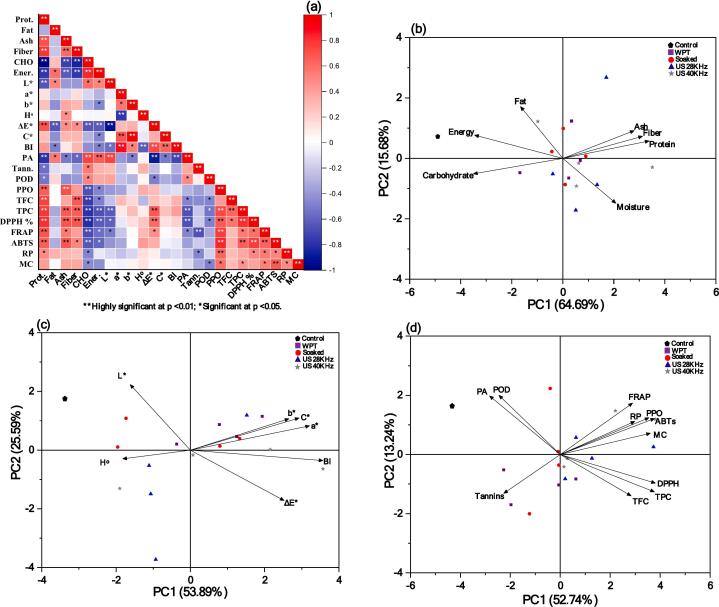
**(a-d).** Correlation Heat map (a); Loading plots of principle components analysis Proximate (b); colour values (c); bioactive components (d). Influence of pretreated germinated quinoa grains followed by drying at various temperatures in hot air dryer.

The link between two variables may be expressed as a correlation coefficient (+1 & −1). If the coefficient is positive, then the variables are positively associated with one another; if it is negative, then the negatively associated with one another. The present study applied Pearson correlation matrices with the correlation coefficient (r) for the samples. Fig. 12**a** shows the relationships between proximate values, colours, and bioactive compounds. There is a strong positive connection between Ash and protein (0.7126) and between fiber (0.7999) and Ash (0.6188) (p < 0.01^**^). A significant negative association were between carbohydrates, protein, ash, and fiber (0.9860, 0.7363, 0.8454) (p < 0.01^**^). Energy were found significantly positive correlated with CHO (0.83330) (p < 0.01^**^), and significantly with Fat (0.5965) (p < 0.05), but negatively correlated with protein (0.7762), Ash (0.6323), and fiber (0.7694) (p < 0.01^**^). Colour data revealed a high positive correlation between Chroma(C*) and a* (0.6908) and b* (0.9892) (p < 0.01^**^), whereas ΔE* was high negatively correlated with L* (0.9339) (p < 0.01^**^). BI had a strong positive correlation with a*, ΔE*, and C* (0.8659), but H° had a high negative correlation (0.7201) (p < 0.01^**^). Our data indicates that phytic acids are high positively correlated with carbohydrates (0.7533) and energy (0.6583), but negatively correlated with protein (0.7908) (p < 0.01^**^). PPO were found high positive correlated with protein (0.6587) and Ash (0.7074), but negatively correlated with carbohydrate (0.6744) (p < 0.01^**^). TPC had a positive correlation with PPO (0.7331) and TFC (0.7215), although negatively correlated with carbohydrate (0.7928), energy (0.6657), and phytic acid (0.6751) (p < 0.01^**^). Significant positive correlations were found between DPPH and protein (0.8190), PPO (0.6775), and TFC (0.8169) (p < 0.01^**^), and TPC (0.5747) (p < 0.05). The correlation between FRAP and protein (0.7815), PPO (0.7665) (p < 0.01^**^), TPC (0.5409), and DPPH (0.5804) (p < 0.05) was significant. ABTs had strong positive correlations with protein, PPO, TPC, DPPH, and FRAP (0.6817, 0.8318, 0.6549, 0.7636, and 0.6364) (p < 0.01^**^). Reducing power correlates positively with PPO (0.6807), significantly with protein (0.5316), DPPH (0.5146), FRAP (0.5403), and ABTS (0.5021) (p < 0.01^**^). Metal chelating was associated positively with ABTs (0.6616) (p < 0.01^**^) and negatively correlated with Carbohydrate (0.5309) and POD (0.5141) (p < 0.05). Also, US 28 kHz and 40 kHz treated quinoa samples had greater bioactive components, antioxidant activity, and APCI (Table 4).

PCA was performed to show the association between pre-treated quinoa before germination and dried at various temperatures. The illustrations shows positive, negative, and no associations between qualities with acute (90◦) or straight (180◦), and right (90◦) angles. PCA proximities (Fig. 12**b**) demonstrated that the first component PC1 (64.69 %) and second component PC2 (15.68 %) principal components contributed 80.37 %) to the showed data's overall variability. An eigenvalue of 4.52 for PC1 explains the largest variability, whereas 0.22 for PC2. PC1 were positively associated with Moisture (0.267), Protein (0.435), Ash (0.360), and Fiber (0.402) and negatively correlated with Fat (0.213), CHO (0.450), and Energy. Moisture (0.535) and Carbohydrate (0.1839) were negatively associated in PC2. In colour values the first component PC1 (52.24 %) with an eigenvalue of 3.77 and the second component PC2 (13.24 %) with an eigenvalue of 0.001 contributed to the depicted data's overall variability (65.48 %) (Fig. 12**c**). PC1 has a positive correlation with a* (0.227), b* (0.255), ΔE* (0.353), C* (0.409), and BI (0.499), but negative correlations with L* (0.227) and H° (0.255). PC2 showed negative H° (0.088), ΔE* (0.514). In bioactive, PC1 (52.74 %) has an eigenvalue of 5.80 and PC2 (13.24 %) has 0.001, with general variability (65.98 %) (Fig. 12**d**). PC1 correlated positively with PPO (0.355), TFC (0.266), TPC (0.354), DPPH (0.358), FRAP (0.333), ABTS (0.393), Reducing power (0.272), and Metal chelating (0.280). PC2 showed negative correlations for tannins (0.280), TFC (0.298), TPC (0.269), and DPPH (0.220) while it was negative co related with Phytic acid (0.266), Tannins (0.213) and POD (0.231). PCA plots most of the negative association occurred by proximities WPT and dried at 70 °C (Fig. 12**b**). In bioactive compounds without pre-treatments at 70 + 60 + 50 °C is responsible for most of the negative contribution reported in (Fig. 12**d)**. Furthermore it shows that FRAP, reducing power, and metal chelating are significantly associated and angularly closer to ABTS activity than DPPH scavenging. The degree to which biplot sample coordinates are similar is directly correlated with their distance from one another. Additionally, ultrasound-pre-treated quinoa germinated samples had greater bioactive components, antioxidant activity, and APCI.

### 3.10 Functional groups

**Fig. 13:**
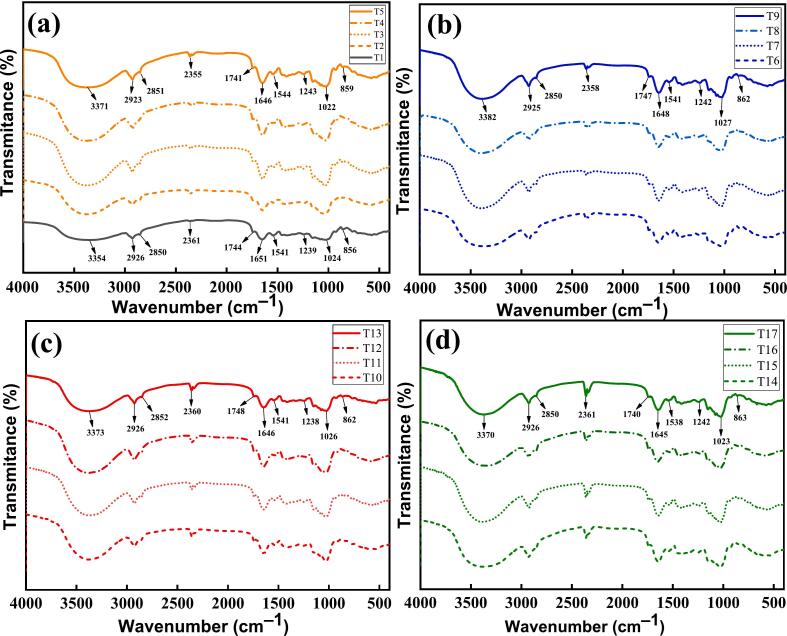
**(a-d).** Functional groups, WPT including the black spectra control (a); soaked (b) US 28 kHz (c); US 40 kHz (d).

Fig. 13**(a-d)** illustrates the infrared spectra of pretreatment-assisted germinated quinoa grains that were dried and germinated; vibrational studies were conducted using a transmission module to examine the sample's functional groups. The wide absorption band at 3354 cm^−1^ to 3382 cm^−1^ in control and pre-treated samples is derived from the vibration of O–H & N–H groups [115]. Similarly, the presence of carboxylic acids or lipids in the flour may have caused the C=O group to be placed between 1741 and 1748 cm^−1^. The stretching of C-H bonds caused by the presence of the CH2-CO-group is ascribed to the bands seen between 2355 and 2361 cm^−1^ [116]. The spectra were similar between the pretreated quinoa grains dried at different temperature, composed of –CH– (2923 to 2926) and –CH_2_– at 2850 to 2852 cm^−1^ and C–OH, found at wave numbers 1022 to 1027 cm^−1^ & –OH 859 to 856 cm^−1^ [116]. The presence of carboxylic acids or lipids in the flour might explain the location of the C=O group at 1741 to 1748 cm^−1^. The bands corresponding to amides I and II, which are 1645 to 1648 cm^−1^ and 1538 to 1544 cm^−1^, respectively, are ascribed. Protein structures include these bands, which correspond to vibration modes of amino acid groups. Also, amino acids were identified by a signal in the C-N vibrational mode, which was seen in the samples at 1238 to 1234 cm^−1^. It is possible to see variations in intensities throughout the samples' spectra, particularly between 800 and 2000 cm^−1^, by examining the spectrograms. In quinoa, chia, and kiwicha flour, as well as in quinoa flour, similar results were observed by García-Salcedo et al. [117]. As the treatment period increases, this suggests that deterioration will continue to worsen. Nevertheless, the germination process did not reveal the creation of any new functional groups. The transmission of signals was greater in the pre-treated samples compared to the control.

### 3.11 Scanning electron microscopy (SEM)

**Fig. 14:**
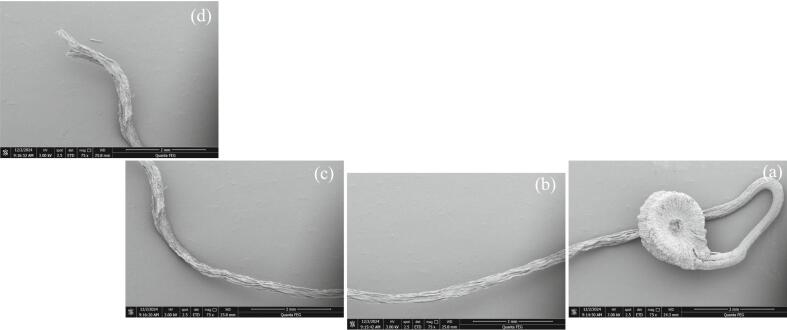
**(a-d).** SEM picture of Germinated quinoa grains.

SEM images depicts the morphology of germinated quinoa grains at various stages of sprouting, showing the external growth of the root (radicle) and surrounding structures at high magnification 75x with scale bar 2 mm (Fig. 14**a-c**).

**Fig. 15:**
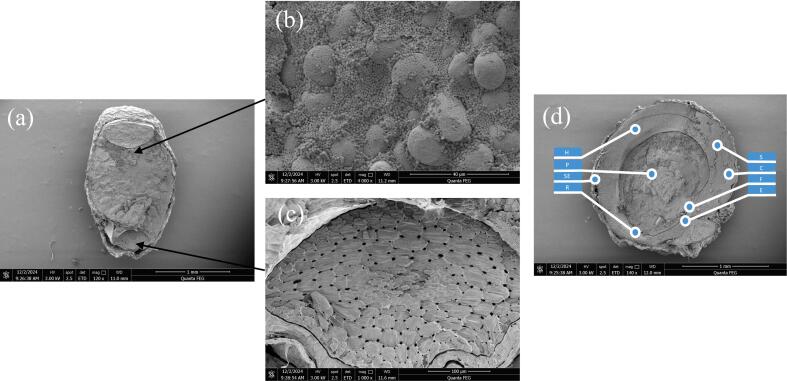
**(a-d).** Scanning electron micrographs of quinoa grains Scanning electron microscope (SEM) of Quinoa grain, longitudinal cross section 120X, 1 mm (a); starch granules and protein bodies 4000X, scale bar (40 µm) (b); embryonic root, 4000X, scale bar (40 µm) (c); grain external view (d).

The quinoa grain is displayed in a longitudinal section, 120x with scale bar 1 mm, providing a clear view of its outer layers and inner composition (Fig. 15**a**). The 4000x with scale bar (40 µm) indicates the highly detailed nature of longitudinal section (Fig. 15**b**). These structures are likely starch granules and protein bodies on the outer surface or just inside the grain. They vary in diameter and appear tightly packed, creating a textured surface (Fig. 15**c**). At 4000x magnification scale bar (40 µm), this image highlights the inner tissue of the quinoa grain. The visible cellular network consists of elongated, tightly packed cells which shows organized, repeating patterns with pores or holes. These cells are likely part of the vascular system or supporting tissues of the embryonic root. This likely represents vascular bundles or cellular structures. The pores may represent air spaces, facilitating gas and nutrient exchange.

**Fig. 16:**
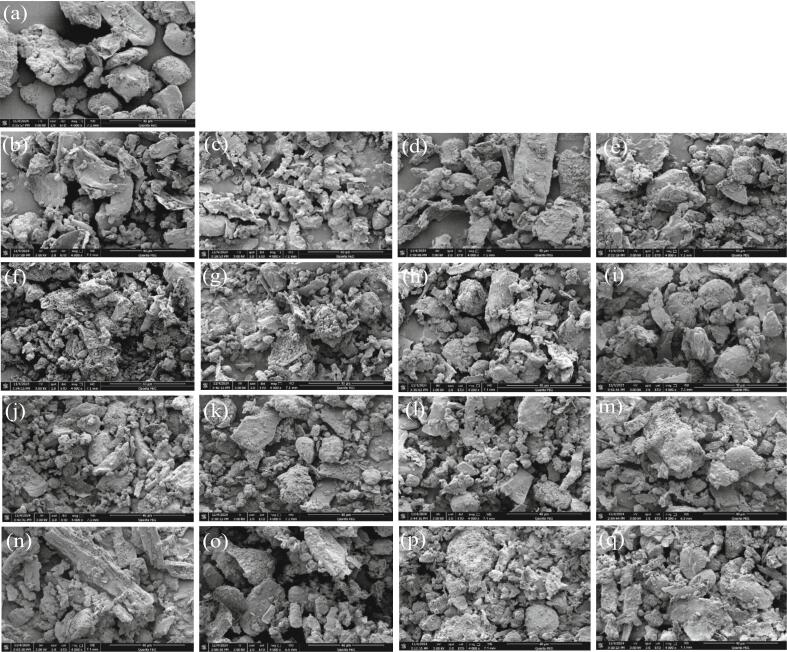
**(a-q).** Scanning electron micrographs of the quinoa samples (a-q) magnification4000x with scale bar (40 µm). Control (a); WPT (b-e), soaked (f-i); US 28 kHz (j-m); US 40 kHz (n-q), germinated samples dried at 50, 60, 70 & combined temperatures.

Finally, the control and pre-treated germinated samples, scanning electron micrographs, 4000x with a scale bar of 40 µm shown in (Fig. 16**a-q**). Fig. 16**a** Control, Fig. 16**b-e** from WPT at 50, 60, 70 & combined temperatures, Fig. 16**f-i** from soaking, Fig. 16**j-m** US 28 kHz, and Fig. 16**n-q** US 40 kHz. The SEM revealed aggregations of germinated quinoa subjected to several temperatures. A film-like material seems to cover and connect the aggregates. A protein matrix is thought by some writers to encase these quinoa starch clumps [[118], [119], [120]]. The shape of starch granules is consistent with prior research, as seen in Fig. 16**b** [[119], [120]]. Having a polygonal form, they were around 1–2 μm in size [121]. Control sample (Fig. 16**a**) showed noticeable differences with respect to pre-treated sample. After pre-treatments especially in US 28 and 40 kHz, the microstructure of was presenting less aggregation between the components. Furthermore Ultrasound treatments, especially at higher frequencies (40 kHz), are the most effective in fragmentation, while without pre-treatment and soaking result in moderate fragmentation. These findings provide further evidence that the starch aggregates are bound to the lipids in addition to being embedded in a protein matrix. In their investigations into the starch-protein-lipid interactions in rice and millet flours, Ye et al. [122] and Annor et al. [123] found comparable results.

## 4 Conclusion

Pre-treatments assisted germination of quinoa grains dried at 60 °C significantly improved the quality profile of quinoa grains. Among evaluated models, page and logarithmic showed the best fit, presenting the highest, R^2^ ≥ 0.9991, X^2^ ≤ 0.0013, RMSE ≤ 0.0022, and RSS ≤ 0.0201. Ultrasonic pre-treated samples US 28 kHz and 40 kHz, dried at 60 °C were found to be appropriate, whereas in overall pretreated germinated sample dried at high temperature 70 °C lead poor quality. The most excellent bioactive compounds and antioxidant capacity increases were achieved at US 40 kHz at 60 °C, yielded the highest APCI of 98.78 %, explaining 80.37 % variability by PCA. Furthermore, ultrasonically pre-treated samples shown a notable decrease of phytic acid and tannin 66.66–82.99 % and 31.48–41.60 %, respectively. These findings emphasize the potential of controlled germination processes, mostly ultrasonic pre-treatment, as effective represents a promising alternative, enhancing antioxidants and may reduce phytic acid in quinoa with improved nutritional attributes. Such studies would demonstrate the feasibility of using this method on a large scale and its possible advantages in creating healthier food products, as well as provide solid evidence in favour of using ultrasonically pre-treated germinated quinoa grain flour as a component in the creation of new foods or nutritional supplements.

## Funding sources

We acknowledge the financial support from National Natural Science Foundation of China (32472381, 32172259), and the Key Research and Development Project of Henan Province (231111111800).

## CRediT authorship contribution statement

**Jabir Khan:** Writing – review & editing, Writing – original draft, Software, Methodology, Formal analysis, Data curation, Conceptualization. **Palwasha Gul:** Visualization, Validation, Data curation, Conceptualization. **Qingyun Li:** Visualization, Validation, Data curation. **Kunlun Liu:** Visualization, Validation, Supervision, Project administration, Methodology, Funding acquisition.

## Declaration of competing interest

The authors declare that they have no known competing financial interests or personal relationships that could have appeared to influence the work reported in this paper.
